# Information-theoretic analysis of multivariate single-cell signaling responses

**DOI:** 10.1371/journal.pcbi.1007132

**Published:** 2019-07-12

**Authors:** Tomasz Jetka, Karol Nienałtowski, Tomasz Winarski, Sławomir Błoński, Michał Komorowski

**Affiliations:** Institute of Fundamental Technological Research, Polish Academy of Sciences, Warsaw, Poland; University of Illinois at Urbana-Champaign, UNITED STATES

## Abstract

Mathematical methods of information theory appear to provide a useful language to describe how stimuli are encoded in activities of signaling effectors. Exploring the information-theoretic perspective, however, remains conceptually, experimentally and computationally challenging. Specifically, existing computational tools enable efficient analysis of relatively simple systems, usually with one input and output only. Moreover, their robust and readily applicable implementations are missing. Here, we propose a novel algorithm, SLEMI—statistical learning based estimation of mutual information, to analyze signaling systems with high-dimensional outputs and a large number of input values. Our approach is efficient in terms of computational time as well as sample size needed for accurate estimation. Analysis of the NF-*κ*B single—cell signaling responses to TNF-*α* reveals that NF-*κ*B signaling dynamics improves discrimination of high concentrations of TNF-*α* with a relatively modest impact on discrimination of low concentrations. Provided R-package allows the approach to be used by computational biologists with only elementary knowledge of information theory.

## Author summary

In light of single-cell, live-imaging experiments understanding of how cells transmit information about identity and quantity of stimuli is incomplete. When exposed to the same stimulus individual cells exhibit substantial cell-to-cell heterogeneity. Besides, stimuli have been shown to regulate temporal profiles of signaling effectors. Therefore, it is, for instance, not entirely clear whether single-cell responses are binary or contain more information about the quantity of stimuli. The above questions resulted in a considerable interest to study cellular signaling within the framework of information theory. Unfortunately, the utilization of the information-theoretic perspective is handicapped in part by the lack of suitable methods that account for multivariate signaling data. Here, we propose a novel algorithm that breaks a considerable computational barrier by allowing the effective information-theoretic analysis of highly-dimensional single-cell measurements. Our approach is computationally efficient, robust and straightforward to use. Moreover, we provide a simple R-package implementation.

This is a *PLOS Computational Biology* Methods paper.

## Introduction

Biochemical descriptions of cellular signaling appear to require quantitative support to explain how complex stimuli (inputs) are translated and encoded in activities of pathway’s effectors (outputs) [1–5]. An attractive approach seems to be offered by probabilistic modeling and information theory [6–10], which provide a mathematical language to describe input-output relationships of complex and stochastic cellular processes. Specifically, the unique perspective of information theory holds a promise of gaining new insights into functional aspects of signaling, as opposed to biochemical and mechanistic descriptions [1, 9, 11–16]. So far, quantification of an overall signaling fidelity and analysis of factors by which it is determined have proven to be useful applications of information theory in studies of cellular signaling [5, 17–19]. Nevertheless, exploring the information-theoretic approach remains conceptually and technically challenging [7, 9, 10]. In particular, for systems with multiple inputs and outputs existing theoretical tools are computationally inefficient and require a large sample size for accurate analysis.

Within information theory, regardless of specific details of a signaling pathway, a signaling system can be considered as an input-output device that measures an input signal, *x*, by eliciting a stochastic output, *Y* [7, 9, 10]. In a typical example, the input, *x*, is the concentration of a ligand, e.g., cytokine, that activates a receptor. The output, *Y*, is an activity of one or more signaling effectors, e.g., of transcription factors quantified over time. As cellular signaling systems are inherently stochastic, the information about the input contained in the output is imprecise and only a limited number of input values can be resolved [6, 15, 20, 21]. To date, a number of studies have experimentally examined fidelity of various signaling systems, e.g., [13, 15, 19, 22–24]. In a typical experiment aimed to quantify fidelity, input values, *x*_1_ ≤ *x*_2_… ≤ *x*_*m*_, ranging from 0 to saturation are considered. In some scenarios, utilization of physiologically arising input concentrations, e.g., morphogen gradients, is also possible [5, 16, 25]. Then, for each input level, *x*_*i*_, cellular responses are quantified in a large number, say *n*_*i*_, of individual cells. Single-cell responses are typically represented as vectors, $y_{l}^{i}$, that contain entries with quantified activities of signaling effectors, where *l* varies from 1 to the number of measured cells, *n*_*i*_. Further, cell-to-cell heterogeneity of responses is simplistically used as a proxy of how reproducible signaling output of an individual cell is. Formally, responses corresponding to each of the inputs, *x*_*i*_, are assumed to follow a probability distribution

$$\begin{array}{c} y_{l}^{i}∼P\left(Y|X=x_{i}\right), \end{array}$$ (1)

which is reconstructed from the data and serves as a model of the single-cell response to the input *x*_*i*_.

Given the above, information theory offers to quantify the overall fidelity of signaling in terms of how many input values, *x*_*i*_, can be resolved based on information contained in the responses. The key factor that determines how many inputs can be resolved is the degree of the overlap between the distributions corresponding to different inputs. For instance, two inputs *x*_1_ and *x*_2_ can be easily resolved if the corresponding output distributions *P*(*Y*|*X* = *x*_1_) and *P*(*Y*|*X* = *x*_2_) are completely distinct, non-overlapping. Then, a given response, *y*, can be assigned without error to the only input for which it can occur. In the scenarios in which *P*(*Y*|*X* = *x*_1_) and *P*(*Y*|*X* = *x*_2_) are overlapping the inputs *x*_1_ and *x*_2_ cannot be resolved as a given response *y* is equally likely to arise for both inputs. The second factor that is essential for formal quantification of the overall fidelity is how frequently the different inputs occur. For illustration, consider a system with three input values, *x*_1_, *x*_2_ and *x*_3_. Suppose, *x*_1_ and *x*_2_ induce very similar output distributions, i.e., *P*(*Y*|*X* = *x*_1_) ≈ *P*(*Y*|*X* = *x*_2_), whereas the input *x*_3_ induces distribution *P*(*Y*|*X* = *x*_3_) that is distinct, non-overlapping, with the first two. How many inputs are on average resolved in this system depends on how frequently different inputs occur. If for instance, *x*_1_ and *x*_3_ occur frequently, e.g., *P*(*x*_1_) = *P*(*x*_3_) ≈ 1/2, whereas, *x*_2_ has a negligible incidence, i.e., *P*(*x*_2_) ≈ 0, the fidelity of signaling allows to resolve two inputs on average. This is because the two inputs that can be resolved occur with high probability. On the other hand, if inputs *x*_1_ and *x*_2_ occur with probability ≈ 1/2, and *x*_3_ has probability close to 0, then only one state can be resolved on average, i.e., concatenation of *x*_1_ and *x*_2_. The frequencies of inputs are taken into account in the form of the input distribution *P*(*X*) = (*P*(*x*_1_), …, *P*(*x*_*m*_)).

The degree of the overlap between the output distributions as well as frequencies of inputs are used to evaluate the overall signaling fidelity in the form of the mutual information

$$\begin{array}{c} MI\left(X,Y\right)=\sum_{i=1}^{m}P\left(x_{i}\right)\int_{R^{d}}P\left(y|X=x_{i}\right)\;{\text{log}}_{2}\frac{P(y|X=x_{i})}{P(y)}dy, \end{array}$$ (2)

where *P*(*y*) is the overall distribution of the output implied by a given distribution of the input, i.e., $P\left(y\right)=\sum_{i=1}^{m}P\left(y|X=x_{i}\right)P\left(x_{i}\right)$. *MI* is expressed in bits and 2^*MI*^can be interpreted as the number of inputs that the system can resolve on average. Definition of mutual information stems from axioms proposed by C. Shannon [26] and can also be written in a more intuitive form of entropy differences, see supporting S1 Text. Selection of the input distribution that is suitable for quantification of information transfer in a specific application can be problematic and provides a degree of arbitrariness. The uniform distribution, i.e., one that gives the same weight to all inputs appear to be a suitable choice in some applications [15]. Alternatively, a most favorable input distribution, *P*(*X*), i.e., the one that maximizes information transfer, can be found. The maximization of mutual information with respect to the input distribution defines the information capacity, C*. Formally,

$$\begin{array}{c} C^{*}=\underset{P(X)}{\text{max}}\;MI\left(X,Y\right). \end{array}$$ (3)

Information capacity is expressed in bits and $2^{C^{*}}$ can be interpreted as the maximal number of inputs that the system can effectively resolve.

In summary, the overall fidelity of a signaling system depends on the degree of the overlap between distributions corresponding to different inputs. The degree of the overlap is translated into the logarithm of the number of resolvable inputs by mutual information, which takes into account how frequently different inputs are transmitted. On the other hand, the information capacity quantifies the logarithm of the number of resolvable inputs under input frequencies that maximize the information transfer. Information capacity, as opposed to mutual information, does not depend on input distribution and therefore may provide a less arbitrary quantification of the overall fidelity.

Existing tools to compute the mutual information and the information capacity [16, 19, 22, 27–29] utilise the data, $y_{l}^{i},$ to construct approximations, $\hat{P}\left(Y|X=x_{i}\right)$, of the output distributions, *P*(*Y*|*X* = *x*_*i*_), for each input, *x*_*i*_. Thereafter, the approximations, $\hat{P}\left(Y|X=x_{i}\right)$, rather than the exact probabilities, *P*(*Y*|*X* = *x*_*i*_), are used for evaluation of the mutual information and information capacity, according to Eqs 2 and 3. The available algorithms differ in the way, in which, the approximations of the output distributions, $\hat{P}\left(y|X=x_{i}\right)$, are constructed. Specifically, Blahut—Arimoto (BA) algorithm [22, 27–29] uses a discrete approximation. All possible values of responses are divided into a finite set of intervals and frequencies of responses falling into the same interval as $y_{l}^{i}$ are used as the approximation of $P(y_{l}^{i}|X=x_{i})$. On the other hand, methods based on the small noise approximation assume Gaussian output with a limited variance [16, 25, 30, 31]. Finally, the approach of [19], following the earlier work [32], uses the k-nearest neighbors (KNN) method, in which continuous approximations of $P(y_{l}^{i}|X=x_{i})$ are constructed based on the distance of $y_{l}^{i}$ to the *k*-th most similar response. Each of the above approaches is practically limited by the dimensionality of the output, *Y*. The BA algorithm can be essentially applied to systems with the one-dimensional output only. On the other hand, for multidimensional outputs, an accurate estimation of *P*(*Y*|*X* = *x*_*i*_) using KNN requires a relatively large sample size [33]. Moreover, KNN demands arbitrary specification of the parameter *k*, which for insufficient data size does not generally guarantee unbiased estimation [32–35], and yields estimation sensitive to algorithm’s settings, i.e., is not parameter-free. Also, KNN based approaches, when used to compute capacity, often require solving computationally expensive optimization problems.

In summary, for multidimensional outputs, the practical difficulty in calculating mutual information, Eq 2, results largely from the lack of methods for accurate estimation of multivariate probability distributions, *P*(*y*|*X* = *x*_*i*_). In addition, calculation of C*, Eq 3, can be problematic, as it requires maximization of the nonlinear function, i.e., *MI*, over the input probability distribution, which can be computationally intense. In Section 1 in S1 Text, we provide more background on information theory and existing computational tools. Here further, we propose an alternative framework, statistical learning estimation of mutual information (SLEMI) that appears to significantly simplify the calculation of the mutual information and the information capacity, especially for systems with high-dimensional outputs and a large number of input values. Moreover, our framework enables simple quantification of the extent to which different inputs can be discriminated.

## Results

The framework to calculate the mutual information and the information capacity proposed here is based on an estimation of the conditional input distribution, *P*(*X*|*Y* = *y*), as opposed to the output distributions *P*(*Y*|*X* = *x*_*i*_) in the existing approaches. Therefore, it bypasses the estimation of, possibly high dimensional, probability densities. Moreover, we show that the use of the conditional input distribution, *P*(*X*|*Y* = *y*), can be combined with an efficient iterative optimization scheme to avoid, potentially problematic, numerical optimization. As a result, the introduced algorithm is computationally and statistically efficient and ensures robust, parameter-free estimation. Besides, we demonstrate that estimation of the conditional input distribution, *P*(*X*|*Y* = *y*), provides a simple way to compute probabilities of correct discrimination between different inputs, which augments insight given by the information capacity. The critical advantage of SLEMI over available approaches is that it allows for efficient and robust analysis of systems with multidimensional outputs and a large number of inputs, which we demonstrate here using numerical test models. Further, we deploy SLEMI to examine single-cell signaling responses of the NF-*κ*B pathway to TNF-*α* stimulation. Our analysis reveales that the NF-*κ*B signaling dynamics improves discrimination of high concentrations of TNF-*α* with a modest impact on discrimination of low concentrations. A robust implementation of the proposed computational tool is provided as the R-package that can be used by computational biologists with only elementary knowledge of information theory.

### Efficient estimation of the mutual information and the information capacity

In contrast to existing approaches, instead of estimating, possibly highly dimensional, conditional output distributions *P*(*Y*|*X* = *x*_*i*_), we propose to estimate the discrete, conditional input distribution, *P*(*x*_*i*_|*Y* = *y*), which is known to be a simpler problem [36, 37]. Estimation of the *MI* using estimates of *P*(*x*_*i*_|*Y* = *y*), denoted here as $\hat{P}\left(x_{i}|Y=y\right)$, is possible as the *MI*, Eq 2, can be alternatively written as [38]

$$\begin{array}{c} MI\left(X,Y\right)=\sum_{i=1}^{m}P\left(x_{i}\right)\int_{R^{d}}P\left(y|X=x_{i}\right)\;{\text{log}}_{2}\frac{P(x_{i}|Y=y)}{P(x_{i})}dy. \end{array}$$ (4)

Although *P*(*Y*|*X* = *x*_*i*_) is still present in the above sum, it represents averaging of the term ${\text{log}}_{2}\frac{P(x_{i}|Y=y)}{P(x_{i})}$ with respect to *P*(*Y*|*X* = *x*_*i*_). The experimental data, however, constitutes a sample from the distribution *P*(*Y*|*X* = *x*_*i*_). The average with respect to distribution *P*(*Y*|*X* = *x*_*i*_) can be, therefore, approximated by the average with respect to data, which is justified by the law of large numbers. Precisely, for a given *P*(*X*) and $\hat{P}\left(x_{i}|Y=y\right)$, *MI* can be approximated with the following formula

$$\begin{array}{c} MI\left(X,Y\right)\approx \sum_{i=1}^{m}P\left(x_{i}\right)\sum_{l=1}^{n_{i}}\frac{1}{n_{i}}{\text{log}}_{2}\frac{\hat{P}\left(x_{i}|Y=y_{l}^{i}\right)}{P(x_{i})}. \end{array}$$ (5)

An estimator $\hat{P}\left(x_{i}|Y=y\right)$, can be built using a variety of Bayesian statistical learning methods. For simplicity and efficiency, here we propose to use logistic regression, which is known to work well in a range of applications [39–43]. In principle, however, other classifiers could also be considered. The logistic regression estimators of *P*(*x*_*i*_|*Y* = *y*) arise from a simplifying assumption that log-ratio of probabilities, *P*(*x*_*i*_|*Y* = *y*) and *P*(*x*_*m*_|*Y* = *y*) is linear. Precisely,

$$\begin{array}{c} \text{log}\;(\frac{P(x_{i}|Y=y)}{P(x_{m}|Y=y)})\approx \alpha_{i}+\beta_{i}^{T}y. \end{array}$$

The above formulation allows fitting the logistic regression equations to experimental data, i.e., finding values of the parameters, *α*_*i*_ and *β*_*i*_ that best represent the data. Once logistic regression parameters are known, estimates $\hat{P}\left(x_{i}|Y=y\right)$ can be constructed. Estimates, $\hat{P}\left(x_{i}|Y=y\right)$, allow, in turn, to calculate mutual information using Eq 5. The use of logistic regression, therefore, constitutes a simple way to evaluate mutual information for multidimensional data without knowledge of *P*(*Y*|*X* = *x*_*i*_). Moreover, fitting the logistic regression equations to experimental data is efficient and available in most statistical software packages. Even though the assumed linear relationship may seem to be an oversimplification, the logistic regression approach has been shown to work exceptionally well in a variety of scenarios and gained broad applicability [36]. *Methods* contain more details on the form and estimation of the logistic regression model.

In addition to the possibility of effective evaluation of the mutual information for models with the multivariate output, *Y*, the use of the logistic regression enables to overcome the potentially problematic numerical, typically gradient, maximization of the mutual information with respect to the input distribution, *P*(*X*), in computations of the information capacity. Precisely, the numerical optimization can be bypassed, by dividing the maximization with respect to the input distribution, *P*(*X*), into two simpler maximization problems, for which explicit solutions exist. Thereafter, a solution of the joint maximization can be obtained from the two explicit solutions in an iterative procedure known as alternate maximization. Compared to gradient optimisation, alternate maximization is superior in terms of computational efficiency, and much less prone to numerical complications. A complete description of the maximization procedure is technically demanding and therefore is provided in *Methods* section. In particular, the algorithm’s pseudo-code is presented in Box 1 therein.

In summary, the use of logistic regression described above allows computing *MI* without estimation of the possibly highly dimensional output distributions *P*(*Y*|*X* = *x*_*i*_). Moreover, it allows for efficient maximization of *MI* without gradient-based methods. In *Methods* and Section 3 in S1 Text, we perform several numerical tests to show how the above design of the algorithm leads to practical benefits in terms of the accuracy of estimation and computational efficiency. Specifically, we demonstrate that SLEMI: (i) provides more accurate estimates than the KNN method, especially for small sample size and highly dimensional output; (ii) delivers robust estimates, insensitive to algorithm’s settings; and (iii) has desired properties in terms of computational cost, especially scales well with the number of input values.

### Probabilities of correct discrimination

The information capacity, C*, tells us how many inputs can be discriminated on average. What it does not tell us directly is which inputs, and to what extent, can be discriminated. Specifically, the same information capacity can result from different patterns of discriminability between input signals. For illustration, consider three inputs, *x*_1_, *x*_2_ and *x*_3_. Assume that inputs *x*_1_ and *x*_2_ induce very similar output distributions, i.e., *P*(*Y*|*X* = *x*_1_) ≈ *P*(*Y*|*X* = *x*_2_), as opposed to *x*_3_ that induces a distinct distribution, *P*(*Y*|*X* = *x*_3_). In such a scenario, the information capacity is approximately 1 bit, as two inputs can be discriminated, i.e., *x*_3_ can be resolved from either of the other two. The capacity of 1 bit would also result from a scenario, in which roles of input values are swapped, say, *P*(*Y*|*X* = *x*_2_) is distinct from the overlapping *P*(*Y*|*X* = *x*_1_) and *P*(*Y*|*X* = *x*_3_). Therefore, it appears that an insight regarding which inputs, and to what extent, can be discriminated can usefully augment computation of the information capacity.

Here, we argue that the extent to which different inputs can be discriminated can be conveniently quantified and visualized using the probability of correct discrimination (PCD) between input pairs. We define the PCD between a pair of input values, *x*_*i*_ and *x*_*j*_, as the fraction of cellular responses that can be assigned correctly to one of the two inputs based on the information contained in the signaling response, *Y*. If the distributions *P*(*Y*|*X* = *x*_*i*_) and *P*(*Y*|*X* = *x*_*j*_) are entirely distinct, knowing the response, *y*, allows assigning each cellular response to the correct input without error. PCD between *x*_*i*_ and *x*_*j*_ is then equal to 1. If, on the other hand, these two distributions are completely overlapping knowing the response, *y*, does not provide any information to assign a cell with a given response to the correct input. In such a case, the discrimination is close to random, yielding half of the cells being assigned correctly, i.e., PCD equals 0.5. Depending on the degree of the overlap, the PCD varies between 0.5 and 1. Further, calculation of PCDs for all input pairs can provide insight regarding which inputs, and to what extent, can be discriminated.

**Fig 1:**
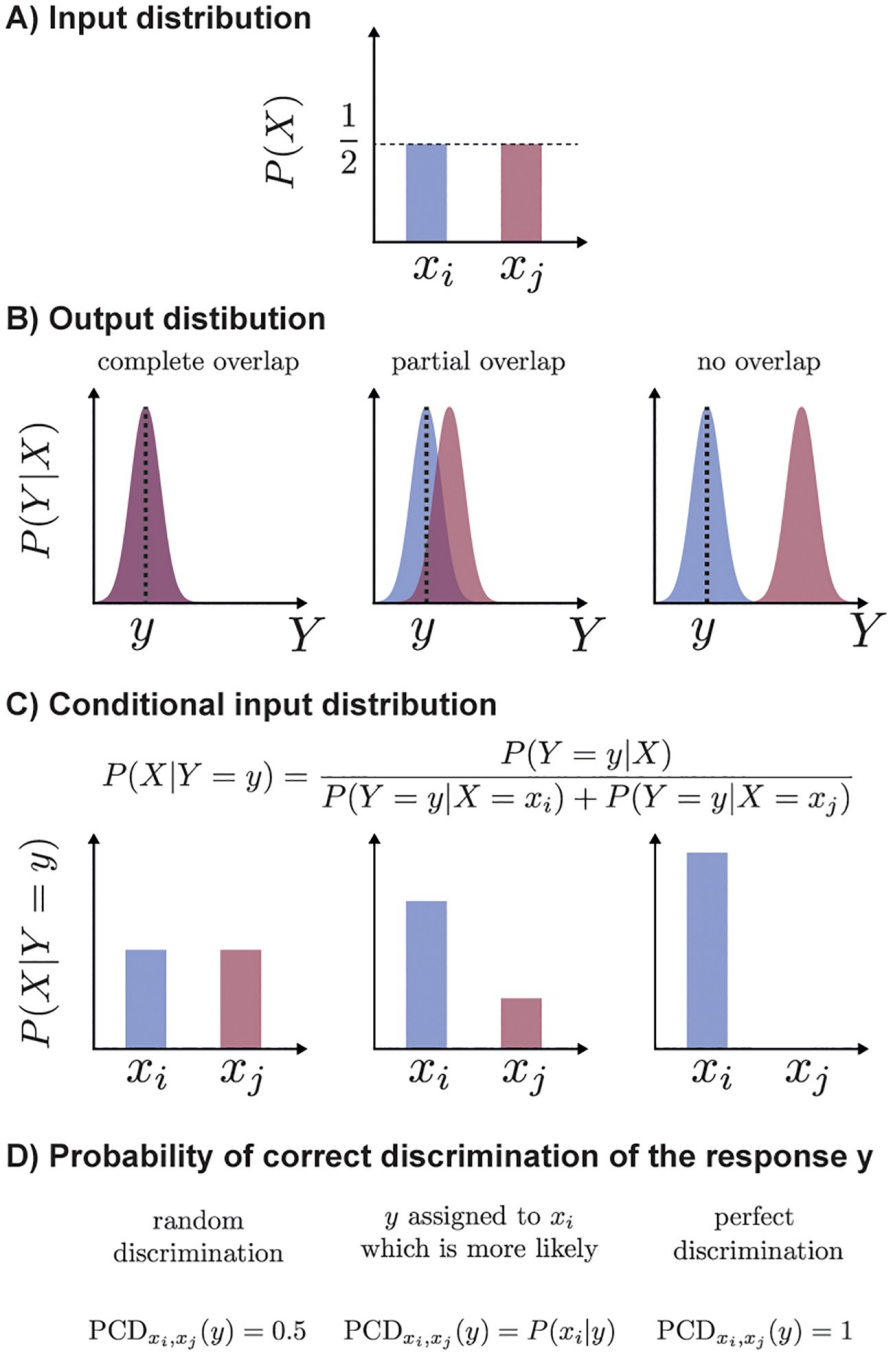
Probabilities of correct discrimination between two inputs, *x*_*i*_ and *x*_*j*_. The input distribution, *P*(*X*) = (1/2, 1/2), visualized in **(A)**, and the conditional output probabilities *P*(*Y*|*X*), presented in **(B)**, can be translated, via the Bayes formula, into conditional input distributions, *P*(*X*|*Y*), visualized in **(C)**. The conditional input distribution, *P*(*X*|*Y*), serves to calculate the probability of correct discrimination of the observation *y*, as shown in **(D)**. Precisely, for any fixed output, *y*, vertical line in (B), the conditional input probability, in (C), *P*(*X*|*Y* = *y*), quantifies how likely it is that *y* was generated by each of the inputs. The probability of correct discrimination of the observation, *y* is given as the maximum of *P*(*x*_*i*_|*Y* = *y*) and *P*(*x*_*j*_|*Y* = *y*). Completely overlapping conditional output probabilities *P*(*Y*|*X*), left column, yield random discrimination as opposed to non-overlapping distributions yielding perfect discrimination, right column. The use of the conditional input distribution, *P*(*X*|*Y*), enables quantification of intermediate scenarios, middle column.

The above intuitions can be mathematically formalized, Fig 1. For formal quantification, in order to treat both inputs equally, we assume that both have the same frequency, *P*(*X*) = (1/2, 1/2), or equivalently that half of the considered cells is stimulated with either of the input values, Fig 1A. How many cells can be assigned correctly depends on the overlap between the distributions *P*(*Y*|*X* = *x*_*i*_) and *P*(*Y*|*X* = *x*_*j*_), Fig 1B. The conditional input distribution, *P*(*x*_*i*_|*Y* = *y*) expresses the probability that a given response *y* was generated by the stimulation level *x*_*i*_, Fig 1C. Equivalently, *P*(*x*_*j*_|*Y* = *y*) is the frequency, at which the given response, *y*, is generated by the stimulation level *x*_*j*_. The probabilities *P*(*x*_*i*_|*Y* = *y*) and *P*(*x*_*j*_|*Y* = *y*) tell us, therefore, how often assignment of the observation *y* to the input *x*_*i*_ and *x*_*j*_, respectively, is correct. To maximize the probability of correct assignment, the response *y* should be assigned to the input for which it is most likely, i.e. to *x*_*i*_ if *P*(*x*_*i*_|*Y* = *y*) ≥ *P*(*x*_*j*_|*Y* = *y*), or to *x*_*j*_, otherwise. Therefore, the observation *y* can be assigned correctly with the probability equal to the maximum of *P*(*x*_*i*_|*Y* = *y*) and *P*(*x*_*j*_|*Y* = *y*). Precisely, the probability of correct discrimination between input *x*_*i*_ and *x*_*j*_ for the response *y*, denoted as $PCD_{x_{i},x_{j}}\left(y\right)$, is calculated as

$$\begin{array}{c} {\text{PCD}}_{x_{i},x_{j}}\left(y\right)=\text{max}\left\{P\left(x_{i}|Y=y\right),P\left(x_{j}|Y=y\right)\right\}, \end{array}$$ (6)

which is visualised in Fig 1D. The average of the above probabilities over cellular responses, $y_{l}^{i}$, corresponding to the input *x*_*i*_ is equal to $\frac{1}{n_{i}}\sum_{l=1}^{n_{i}}PCD_{x_{i},x_{j}}\left(y_{l}^{i}\right)$ and quantifies the average probability of correct discrimination of responses induced by the input *x*_*i*_. Then, the overall probability of correct discrimination between *x*_*i*_ and *x*_*j*_ is given as

$$\begin{array}{c} {\text{PCD}}_{x_{i},x_{j}}=\frac{1}{2}\frac{1}{n_{i}}\sum_{l=1}^{n_{i}}PCD_{x_{i},x_{j}}\left(y_{l}^{i}\right)+\frac{1}{2}\frac{1}{n_{j}}\sum_{l=1}^{n_{j}}PCD_{x_{i},x_{j}}\left(y_{l}^{j}\right). \end{array}$$ (7)

In summary, the probability of correct discrimination between inputs *x*_*i*_ and *x*_*j*_, ${\text{PCD}}_{x_{i},x_{j}}$, quantifies the fraction of cellular responses that can be correctly assigned to either of the inputs based on the output, *Y*. Therefore, the calculation of PCDs for all pairs of input values reveals the extent to which different inputs are discriminated.

From the computational perspective, PCDs are defined in terms of the conditional input probabilities, *P*(*x*_*i*_|*Y* = *y*), Therefore, similarly to the mutual information, these can be calculated using logistic regression. In *Methods* we provide practical details on how to compute PCDs. In the analysis of the NF-*κ*B signaling data, presented below, we show how quantification of PCDs along with computation of the information capacity C* helps to provide insight regarding how signaling dynamics increases overall signaling fidelity.

### Signaling dynamics of the NF-*κ*B system strongly improves discrimination of only high TNF-*α* concentrations

**Fig 2:**
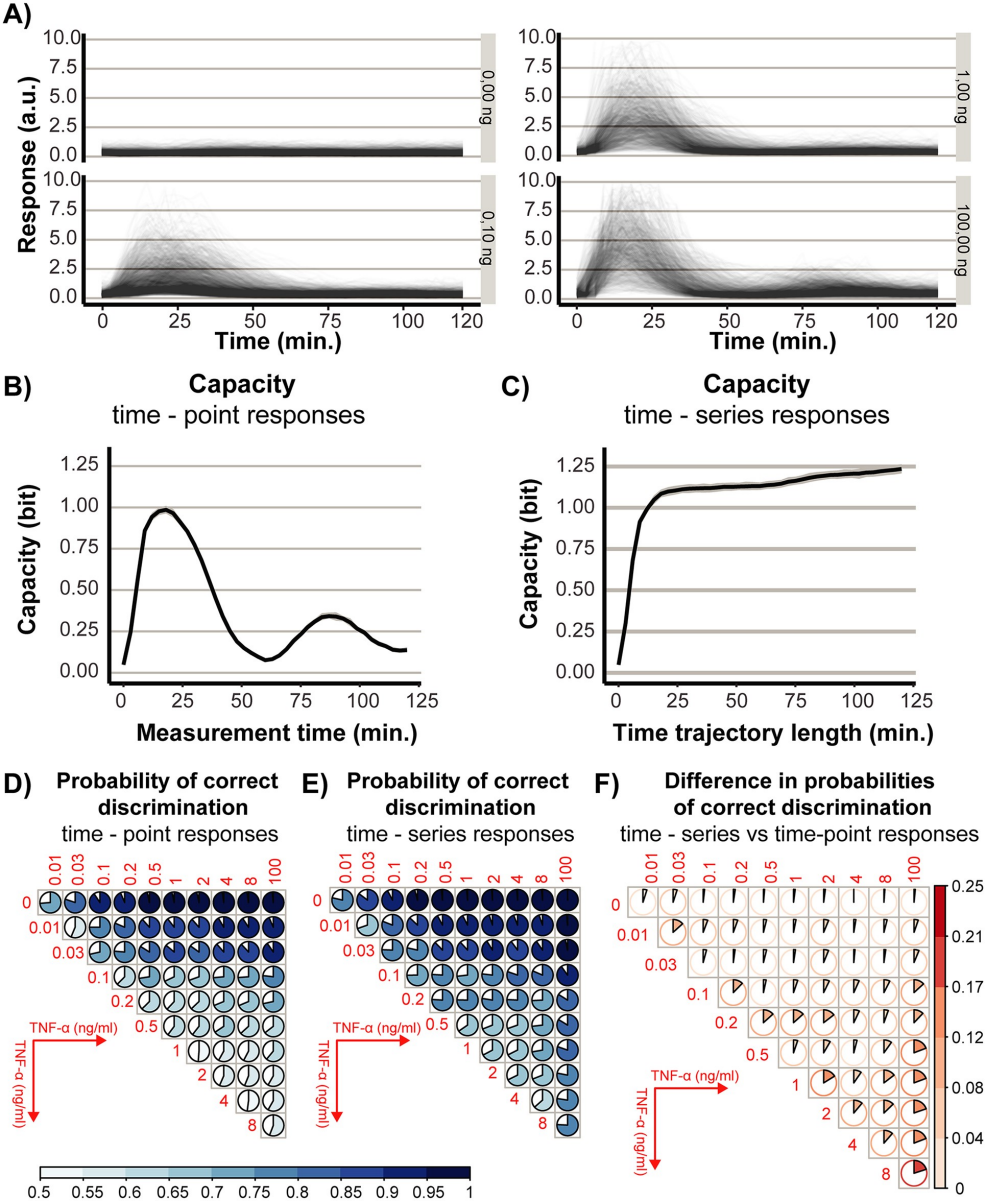
Information-theoretic analysis of the NF-*κ*B responses to TNF-*α* stimulation. **(A)** Temporally resolved responses of individual cells to selected concentrations of TNF-*α*. The panel corresponds to Fig. IV in S1 Text. **(B)** The information capacity as a function of time for time-point responses. **(C)** As in (B) but for time-series responses. **(D)** Probabilities of the correct pairwise discrimination between TNF-*α* concentrations for time-point responses at 21 minutes. The color filled fraction of the circle marks the probability of correct discrimination. **(E)** The same as in (D) but for time-series responses. **(F)** Differences between probabilities in (D) and (E). Modeling details: Uncertainties of estimates (grey ribbons in B and C) were obtained by bootstrapping 80% of data (repeated 100 times). Probabilities in (D) and (E) present mean of 50 bootstrap re-sampling.

NF-*κ*B pathway is one of the key biochemical circuits involved in the control of the immune system [44–46]. It is also one of the first cellular signaling systems studied within the framework of information theory [22]. So far, several papers examined its dose dependency, e.g., [12, 44, 47] and quantified its information capacity, e.g., [13, 19, 22]. Interestingly, response dynamics have been shown to have greater signaling capacity compared to time-point, non-dynamic, responses [13, 19]. To demonstrate what benefits result from efficient calculation of the information capacity and of the probabilities of correct discrimination, we have measured NF-*κ*B responses ($y_{l}^{i}$’s in our notation) to a range of 5 minutes pulses of TNF-*α* concentrations (*x*_*i*_’s), in single—cells, using life confocal imaging. Experimental methods are described in Sections 4.1—4.3 in S1 Text. Fig 2A shows temporally resolved responses to representative four concentrations, whereas Fig. IV, in S1 Text, to all ten considered concentrations. Further, we used the data to calculate the information capacity between TNF-*α* concentration and cellular response for two different scenarios: time-point and time-series. Precisely, for the time-point scenario, we considered single-cell measurements at each time-point separately. In this case, the signaling output of an individual cell at a given time, *y*, is represented by a single number, which is different for different time-points. On the other hand, for the time-series scenario, we considered single-cell measurements from the beginning of the experiment until an indicated time. In this case signaling output of an individual cell, *y*, is a vector corresponding to a time window from 0 till a given time. Fig 2B and 2C show information capacity as the function of time for the time-point and time-series scenario, respectively.

For the time-point scenario, the capacity increases at early times and reaches the maximum of ≈ 1 bit at ≈ 20 minutes, which coincides with the time of maximum response of trajectories shown in Fig 2A. Interestingly, the second peak of information transfer, of ≈ 0.3 bits, appears at ≈ 90 minutes. Inspection of the response trajectories, Fig 2A, around 90 minutes allows for an interpretation of the second peak. Comparison of the response trajectories corresponding to 1 and 100 ng/ml of TNF- *α*, Fig 2A, indicates the emergence of a fraction of cells that exhibit a second peak in response to the highest considered concentration. The second peak is reminiscent of the oscillatory behavior that is typical for the NF-*κ*B pathway when exposed to continuous, as opposed to 5 minutes, stimulation [44, 48–50]. The second peak in response trajectories carries some information about TNF-*α* and, therefore, contributes to the second peak of information transfer.

For the time-series scenario the capacity also rapidly increases at early times to reach 1 bit at 20 minutes. For later times, the capacity continues to increase but at a much slower rate, with a modest acceleration around 70 minutes, to reach ≈ 1.3 bits at the end of the experiment. As the information contained in shorter time-series is contained in longer time-series, the capacity does not decrease. An increase in the capacity in a given time interval indicates that new information is arriving. Analogously, a time interval with a plateau demonstrates the lack of new information being transmitted at times of that interval. Therefore, our analysis demonstrates that most of the information, ≈ 1 bit, is transferred relatively early, i.e., within the first 20 minutes after stimulation. Later times provide ≈ 0.3 bits of new information. In Section 4.5 in S1 Text we use the method proposed in [51] to further examine the redundancy of information contained in responses at individual time-points.

The higher information content of the time-series poses the question: what type of information is contained in the time-series responses that is not encapsulated in the time-point responses? The information capacity *per se*, being an overall measure of signaling fidelity, does not tell us to what extent specific inputs can be discriminated. Therefore, the information capacity alone cannot reveal which inputs gain discriminability due to signaling dynamics. In order to address the above question in detail, we have calculated the probabilities of correct discrimination, PCDs, for each pair of concentrations, *x*_*i*_, *x*_*j*_. Analogously as in the computation of the capacity, we have considered the time-point and time-series scenarios. PCDs for the two scenarios are shown in Fig 2D and 2E. The filled fractions of the pies mark the PCDs between the corresponding pairs of concentrations, i.e., the fractions of cells that can be assigned to the correct input. The plots, primarily reveal how the time-point and time-series capacities of 1 and 1.3 bits, respectively, translate into discrimination between pairs of inputs. They show that concentrations far apart can be easily discriminated in both scenarios. For instance, PCDs between 0 and 100 ng/ml are close to 1. The discrimination of closer concentrations is more difficult. For instance, in both scenarios, PCDs between 0 and 0.01 ng/ml are approximately 0.75, which corresponds to 0.25 probability of incorrect assignment.

Interestingly, for time-point responses, PCDs between pairs of concentrations ≥ 1 ng/ml are close to 0.5, which implies lack of discriminability. This is, however, not the case for time-series responses where PCDs are considerably greater than 0.5. Comparison of PCDs between the two scenarios reveals, therefore, which states gain discriminability due to signaling dynamics. Fig 2F presents differences between PCDs for time-series and time-points. The increase in discriminability resulting from signaling dynamics is particularly striking for high concentrations, say > 1 ng/ml. These concentrations are only weakly discriminated for the time-point scenario. Certain low concentrations also gain discriminability, e.g., 0.01 and 0.03. However, overall, the increase in discriminability is not so significant for low concentrations as these are relatively well discriminated in the time-point scenario.

The above analysis yields similar conclusions to these presented in [19]. Here, we used 5 minutes TNF-*α* as opposed to continuous lipopolysacharide stimulation in [19]. Also, we used a more complete, higher dimensional, response trajectories, which allowed to plot the temporal profile of information transfer. Our analysis of PCDs complemented calculation of the capacity by revealing discriminability between different inputs. Indeed, values far apart are discriminated virtually without error. Closer concentration gain discriminability due to dynamics of signaling, whereas the gain is particularly strong for high concentrations.

### R-package

Our algorithm is available as robustly implemented R-package, SLEMI. It is designed to be used by computational biologists with a limited background in information theory. It includes functions to calculate mutual information, information capacity and probabilities of correct discrimination. These functions take as the argument a data frame, data, containing signaling responses stored in the following form

$$\begin{array}{ccccc} \text{input} & \text{output}\;1 & \text{output}\;2 & \text{output}\;3 & \ldots \\ \begin{array}{c} n_{1}\{\begin{array}{c} x_{1}\\ \vdots \\ x_{1} \end{array}\\ n_{2}\{\begin{array}{c} x_{2}\\ \vdots \\ x_{2} \end{array}\\ \vdots \\ n_{m}\{\begin{array}{c} x_{m}\\ \vdots \\ x_{m} \end{array} \end{array} & \begin{array}{c} \begin{array}{c} y_{1,1}^{1}\\ \vdots \\ y_{n_{1},1}^{1} \end{array}\\ \begin{array}{c} y_{1,1}^{2}\\ \vdots \\ y_{n_{2},1}^{2} \end{array}\\ \vdots \\ \begin{array}{c} y_{1,1}^{m}\\ \vdots \\ y_{n_{m},1}^{m} \end{array} \end{array} & \begin{array}{c} \begin{array}{c} y_{1,2}^{1}\\ \vdots \\ y_{n_{1},2}^{1} \end{array}\\ \begin{array}{c} y_{1,2}^{2}\\ \vdots \\ y_{n_{2},2}^{2} \end{array}\\ \vdots \\ \begin{array}{c} y_{1,2}^{m}\\ \vdots \\ y_{n_{m},2}^{m} \end{array} \end{array} & \begin{array}{c} \begin{array}{c} y_{1,3}^{1}\\ \vdots \\ y_{n_{1},3}^{1} \end{array}\\ \begin{array}{c} y_{1,3}^{2}\\ \vdots \\ y_{n_{2},3}^{2} \end{array}\\ \vdots \\ \begin{array}{c} y_{1,3}^{m}\\ \vdots \\ y_{n_{m},3}^{m} \end{array} \end{array} & \begin{array}{c} \cdots \end{array} \end{array}.$$ (8)

Each row, *l*, represents a single cell. The first column contains stimulation levels, *x*_*i*_. Further columns contain entries corresponding to measurements of cellular output, $y_{l,d}^{i}$, e.g., subsequent elements of a time-series.

Upon download with the function install_github() of the ‘devtools’ package

install_github(“sysbiosig/SLEMI”)

the considered information-theoretic measures can be calculated by running

mi_logreg_main(data)

for *MI*, with uniformly distributed inputs;

capacity_logreg_main(data)

for information capacity C*; and

prob_discr_pairwise(data)

for probabilities of correct discrimination.

More details on installation and applicability are provided in the package’s *User Manual*. A step-by-step *Testing Procedures* file is also available to assist with running essential functions. The package includes the NF-*κ*B dataset as well as scripts to reproduce Fig 2. Computations needed to plot each panel of the figure, without bootstrap, do not exceed several minutes on a regular laptop.

## Discussion

Building upon existing approaches, our framework considerably simplifies information-theoretic analysis of multivariate single-cell signaling data. It benefits from a novel algorithm, which is based on the estimation of the discrete input distribution as opposed to the estimation of continuous output distributions. Conveniently, the algorithm does not involve numerical gradient optimization. These factors result not only in short computational times but, also, in relatively low sample sizes needed to obtain accurate estimates. Therefore, our framework is particularly suitable to study systems with high dimensional outputs and a large number of input values. Also, the approach relates the information capacity to the probability of discrimination between different input values.

Information theory seems to offer useful tools to provide a better understanding of how cells transmit information about identity and quantity of stimuli, and further how signaling systems enable cells to perform complex functions. Such tools might have a fair potential to successfully augment more traditional approaches. The latter provided a relatively good understooding of the overall molecular and biochemical mechanisms how individual cells transmit signals to effectors [1]. However, we lack understanding of how the stimuli are translated into distinct responses and, hence, how to effectively control cellular processes and decisions [1, 3, 15]. Specifically, the induction of distinct responses in individual cells by means of biochemical interventions in non-trivial settings is most often problematic [52–54]. Results of our work appear to contribute a relevant tool to apply information-theoretic analysis to more complex, particularly highly multivariate, data sets on signaling systems than achievable with available approaches. The multivariate aspect seems to be particularly relevant given the complexity of cellular processes. Hopefully, current and future development of multivariate single-cell measurement techniques, accompanied by computational tools, will enable utilization of the information-theoretic perspective in more complex scenarios. These in turn appear to have a potential to provide a comprehensive insight into how cells can derive a variety of distinct outputs from complex inputs using noisy, cross-wired and dynamic signaling pathways.

## Methods

### Logistic regression

The logistic regression model is the state-of-the-art statistical method to estimate the probability of a given observation, i.e., data vector, *y*, belonging to one of the *m* considered classes. In the setting of the paper, classes correspond to input values and observations to signaling responses. The method assumes that the overall frequencies of observations belonging to each class are described by a probability distribution. In the paper’s setting, these probabilities correspond to the input distribution *P*(*X*) = (*P*(*x*_1_), …, *P*(*x*_*m*_)).

The method is based on the assumption that for a given *P*(*X*), the ratio of the probability of the observation *y* belonging to class *i* to the same probability for the class *m* is linear with respect to *y*. Precisely, denoting the logistic regression estimate of the probability of a given observation, *y*, belonging to the class *i* as ${\hat{P}}_{lr}\left(x_{i}|Y=y;P\left(X\right)\right)$, the above assumption writes as follows

$$\begin{array}{ccc} \text{log}\;(\frac{{\hat{P}}_{lr}\left(x_{1}|Y=y;P\left(X\right)\right)}{{\hat{P}}_{lr}\left(x_{m}|Y=y;P\left(X\right)\right)}) & \approx & \alpha_{1}+\beta_{1}^{T}y,\\ & \vdots & \\ \text{log}\;(\frac{{\hat{P}}_{lr}\left(x_{i}|Y=y;P\left(X\right)\right)}{{\hat{P}}_{lr}\left(x_{m}|Y=y;P\left(X\right)\right)}) & \approx & \alpha_{i}+\beta_{i}^{T}y,\\ & \vdots & \\ \text{log}\;(\frac{{\hat{P}}_{lr}\left(x_{m-1}|Y=y;P\left(X\right)\right)}{{\hat{P}}_{lr}\left(x_{m}|Y=y;P\left(X\right)\right)}) & \approx & \alpha_{m-1}+\beta_{m-1}^{T}y \end{array}$$ (9)

and $\sum_{i=1}^{m}{\hat{P}}_{lr}\left(x_{i}|Y=y;P\left(X\right)\right)=1$. Given the linear form of the above equations, for a data set given as Eq 8, estimation of the parameters *α*_*i*_ and *β*_*i*_ can be done efficiently with state-of-the-art methods [36, 55]. Further, the above equations imply that estimates, ${\hat{P}}_{lr}\left(x_{i}|Y=y;P\left(X\right)\right)$, can be explicitly written as

$$\begin{array}{ccc} {\hat{P}}_{lr}\left(x_{1}|Y=y;P\left(X\right)\right) & = & \frac{\text{exp}\;(\alpha_{1}+\beta_{1}^{T}y)}{1+\sum_{r=1}^{m-1}\text{exp}\;(\alpha_{r}+\beta_{r}^{T}y)},\\ & \vdots & \\ {\hat{P}}_{lr}\left(x_{i}|Y=y;P\left(X\right)\right) & = & \frac{\text{exp}\;(\alpha_{i}+\beta_{i}^{T}y)}{1+\sum_{r=1}^{m-1}\text{exp}\;(\alpha_{r}+\beta_{r}^{T}y)},\\ & \vdots & \\ {\hat{P}}_{lr}\left(x_{m-1}|Y=y;P\left(X\right)\right) & = & \frac{\text{exp}\;(\alpha_{m-1}+\beta_{m-1}^{T}y)}{1+\sum_{r=1}^{m-1}\text{exp}\;(\alpha_{r}+\beta_{r}^{T}y)},\\ {\hat{P}}_{lr}\left(x_{m}|Y=y;P\left(X\right)\right) & = & \frac{1}{1+\sum_{r=1}^{m-1}\text{exp}\;(\alpha_{r}+\beta_{r}^{T}y)}. \end{array}$$ (10)

### Maximization algorithm to compute capacity

Below, we describe maximization of the mutual estimation, *MI*, with respect to the input distribution *P*(*X*), using the so-called alternate optimization, which bypasses gradient optimization. The proposed algorithm is largely based on the original Blahut-Arimoto approach [27, 28]. It is adapted to work with logistic regression and, hence, with continuous and multidimensional output, *Y*. The provided Lemmas are minor modifications of the original Blahut-Arimoto results to account for continuous and multidimensional output.

The algorithm is based on the following five key components. One, the maximization of mutual information with respect to input distribution, *P*(*X*), is replaced with a double maximization, i.e., maximization with respect to the input distribution, *P*(*X*), and with respect to tailored auxiliary function, *Q*(*X*|*Y*). The function *Q*(*X*|*Y*) is introduced to dissect the effects of the input distribution, *P*(*X*), and the conditional input distribution, *P*(*Y*|*X*), on the mutual information, *MI*. Two, explicit solutions of the individual maximizations are found. Three, the individual maximizations are combined in an iterative procedure to provide the solution of the joint maximization. Four, integrals involved in the evaluation of the optimal solutions of individual maximizations are computed through averaging with respect to data. Five, logistic regression is used to evaluate optimal *Q*(*X*|*Y*) at each step of the iterative procedure. Each of the above five elements is described in detail below, and the complete algorithm is summarized in Box 1.

#### Information capacity as a double maximization problem

The first element of the proposed approach involves replacing the maximization in the capacity definition, Eq 3, with the double maximization. Precisely, it can be shown (Lemma 1 in S1 Text) that in the setting of Eq 1, the capacity, C*, Eq 3, can be written as

$$\begin{array}{c} C^{*}=\underset{P(X)}{\text{max}}\underset{Q(X|Y)}{\text{max}}J\left(P\left(X\right),Q\left(X|Y\right)\right), \end{array}$$ (11)

where *Q*(*X*|*Y*), for a given *Y* = *y*, is a discrete probability distribution with respect to *X*, whereas *J* is an auxiliary function defined as

$$\begin{array}{c} J\left(P\left(X\right),Q\left(X|Y\right)\right)=\sum_{i=1}^{m}P\left(x_{i}\right)\int_{R^{d}}P\left(y|X=x_{i}\right)\;{\text{log}}_{2}\frac{Q(x_{i}|y)}{P(x_{i})}dy. \end{array}$$ (12)

The function *J* is introduced for technical convenience and was obtained from the mutual information, Eq 4, by replacing *P*(*X*|*Y*) with *Q*(*X*|*Y*). To prove Lemma 1 in S1 Text, we also shows that for *Q*(*X*|*Y*) = *P*(*X*|*Y*), the function *J* is maximised and equal to the mutual information. It is a generalisation of Theorem 1a of [27] to work for systems with continuous and multidimensional output, *Y*.

#### Individual maxima can be found explicitly

Compared to the single maximization problem with respect to *P*(*X*) in Eq 3, the double maximization in Eq 11 with respect to *P*(*X*) and *Q*(*X*|*Y*) has the advantage that the optimal solutions of the individual optimisation problems can be found analytically.

Precisely, the individual maximizations

$$\begin{array}{c} P^{*}\left(X;Q\left(X|Y\right)\right)=\underset{P(X)}{\text{arg}\;\text{max}}\;J\left(P\left(X\right),Q\left(X|Y\right)\right), \end{array}$$ (13)

$$\begin{array}{c} Q^{*}\left(X|Y;P\left(X\right)\right)=\underset{Q(X|Y)}{\text{arg}\;\text{max}}\;J\left(P\left(X\right),Q\left(X|Y\right)\right) \end{array}$$ (14)

have explicit solutions. The solution of the maximization in Eq 13 can be found using Lagrange multipliers. Lemma 2 in S1 Text shows that for a given *Q*(*X*|*Y*) the optimal value of

$$\begin{array}{c} \underset{P(X)}{\text{max}}\;J\left(P\left(X\right),Q\left(X|Y\right)\right) \end{array}$$ (15)

is achieved by

$$\begin{array}{c} P^{*}\left(x_{i};Q\left(X|Y\right)\right)=\frac{\text{exp}(D_{i}\left(Q\right))}{\sum_{r=1}^{m}\text{exp}\left(D_{r}\left(Q\right)\right)}, \end{array}$$ (16)

where

$$\begin{array}{c} D_{i}\left(Q\right)=\int_{R^{d}}P\left(y|X=x_{i}\right)\;{\text{log}}_{2}Q\left(x_{i}|y\right)dy. \end{array}$$ (17)

The above is similar to the solution presented in the conventional BA algorithm [27] but accounts for continuous output *Y*.

Solution of the maximization in Eq 14 is a well established result provided in [27] as Theorem 1, which is used here in an unchanged form. Precisely, for a given *P*(*X*), Eq 14 has the explicit solution

$$\begin{array}{c} Q^{*}\left(x_{i}|y;P\left(X\right)\right)=\frac{P\left(x_{i}\right)P\left(y|X=x_{i}\right)}{\sum_{r=1}^{m}P\left(x_{r}\right)P\left(y|X=x_{r}\right)}. \end{array}$$ (18)

#### Alternate maximization

Further, similarly as in the BA approach, we propose to combine the above solutions, i.e., Eqs 16 and 18, in the so called alternate maximization (AM) iterative procedure to deliver the solution of the joint maximization of Eq 11. Precisely, in an initial step, arbitrary *P*(*X*) and *Q*(*X*|*Y*), denoted here as *P*^(0)^(*x*_*i*_), *Q*^(0)^(*x*_*i*_|*y*), respectively, are assumed. Thereafter, at each step indexed by *k* new *P*(*X*) and *Q*(*X*|*Y*), denoted as *P*^(*k*)^(*x*_*i*_), *Q*^(*k*)^(*x*_*i*_|*y*), are found. Precisely, *P*^(*k*)^(*x*_*i*_) and *Q*^(*k*)^(*x*_*i*_|*y*) are set to optimal solutions of the individual maximization problems of Eqs 13 and 14 given previous values *P*^(*k*−1)^(*x*_*i*_), *Q*^(*k*−1)^(*x*_*i*_|*y*), i.e.,

$$\begin{array}{c} P^{\left(k\right)}\left(x_{i}\right)=P^{*}\left(x_{i};Q^{\left(k-1\right)}\left(x_{i}|y\right)\right), \end{array}$$ (19)

$$\begin{array}{c} Q^{\left(k\right)}\left(x_{i}|y\right)=Q^{*}\left(x_{i}|y;P^{\left(k\right)}\left(x_{i}\right)\right). \end{array}$$ (20)

By Lemma 2 the capacity, C*, is then approximated as

$$\begin{array}{c} C^{\left(k\right)}=J\left(P^{\left(k\right)}\left(X\right),Q^{\left(k\right)}\left(X|Y\right)\right)=\sum_{i=1}^{m}P^{\left(k\right)}\left(x_{i}\right)(D_{i}\left(Q^{\left(k\right)}\right)-{\text{log}}_{2}P^{\left(k\right)}\left(x_{i}\right)). \end{array}$$ (21)

In Lemma 4, we generalise the result of [28], and we show that for systems with continuous output *Y*, the iterative scheme converges to the solution of the joint maximization problem, Eq 11, precisely

$$\begin{array}{c} P^{\left(k\right)}\left(x_{i}\right)\overset{k\to \infty }{\to }P^{*}\left(x_{i}\right) \end{array}$$

and

$$\begin{array}{c} C^{\left(k\right)}\overset{k\to \infty }{\to }C^{*}. \end{array}$$

#### Integration through averaging

For a practical implementation of the above AM method, solutions of the individual maximizations problems need to be found for a given data set. Precisely, Eqs 12, 16 and 18 need to be numerically evaluated, which is seemingly problematic, as these equations depend on the unknown conditional probabilities *P*(*Y*|*X*). Therefore, we propose a strategy to evaluate Eqs 12, 16 and 18 without knowledge of *P*(*Y*|*X*). Primarily, we show below that integration with respect to *y*, in Eqs 12 and 17, can be performed without knowledge of *P*(*y*|*X* = *x*_*i*_). Thereafter, we demonstrate how *Q*^(*k*)^(*x*_*i*_|*y*) can be computed.

Eqs 12 and 17 involve integrals

$$\begin{array}{c} \int_{R^{d}}P\left(y|X=x_{i}\right)\;{\text{log}}_{2}\frac{Q(x_{i}|y)}{P(x_{i})}dy \end{array}$$

and

$$\begin{array}{c} \int_{R^{d}}P\left(y|X=x_{i}\right)\;{\text{log}}_{2}Q\left(x_{i}|y\right)dy, \end{array}$$

respectively, that denote expectations with respect to the distribution *P*(*Y*|*X* = *x*_*i*_), i.e., $E_{P(Y|X=x_{i})}(\cdot )$. The law of large numbers implies that expectations can be approximated solely based on a sample from the distribution *P*(*Y*|*X* = *x*_*i*_). Indeed, if only the number of observations in experimental data is large enough, the average computed based on the sample approximates the expectation, leading to

$$\begin{array}{ccc} \int_{R^{d}}P\left(y|X=x_{i}\right)\;{\text{log}}_{2}\frac{Q(x_{i}|y)}{P(x_{i})}dy & = & E_{P(Y|X=x_{i})}({\text{log}}_{2}\frac{Q(x_{i}|Y)}{P(x_{i})})\approx \frac{1}{n_{i}}\sum_{l=1}^{n_{i}}{\text{log}}_{2}\frac{Q(x_{i}|y_{l}^{i})}{P(x_{i})}, \end{array}$$ (22)

$$\begin{array}{ccc} \int_{R^{d}}P\left(y|X=x_{i}\right)\;{\text{log}}_{2}\;Q\left(x_{i}|y\right)dy & = & E_{P(Y|X=x_{i})}\;{\text{log}}_{2}\;Q\left(x_{i}|Y\right)\approx \frac{1}{n_{i}}\sum_{l=1}^{n_{i}}{\text{log}}_{2}\;Q\left(x_{i}|y_{l}^{i}\right). \end{array}$$ (23)

The above shows, that the integration of Eqs 12 and 17, indeed does not require explicit knowledge of *P*(*y*|*X* = *x*_*i*_). Below, we show that computation of Q*(*x*_*i*_|*y*; *P*(*X*)) in Eq 18 does not require evaluation of *P*(*y*|*X* = *x*_*i*_) either.

#### Incorporation of logistic regression

The Bayes conditional probability formula

$$\begin{array}{c} P\left(x_{i}|Y=y\right)=\frac{P\left(x_{i}\right)P\left(y|X=x_{i}\right)}{\sum_{r=1}^{m}P\left(x_{r}\right)P\left(y|X=x_{r}\right)} \end{array}$$

implies that, for a given *P*(*X*), Q*(*x*_*i*_|*y*; *P*(*X*)) defined by Eq 18 is equivalent to *P*(*x*_*i*_|*Y* = *y*), i.e.,

$$\begin{array}{c} Q^{*}\left(x_{i}|y;P\left(X\right)\right)=P\left(x_{i}|Y=y\right). \end{array}$$ (24)

Therefore, for a given *P*(*X*) finding Q*(*x*_*i*_, *y*; *P*(*X*)) is equivalent to finding *P*(*x*_*i*_|*Y* = *y*) and, therefore, *P*(*y*|*X* = *x*_*i*_) is not required. As discussed eariler, approximation of *P*(*x*_*i*_|*Y* = *y*) is a classification problem in the statistical learning theory [36] and here we propose to approximate *P*(*x*_*i*_|*Y* = *y*) and, hence, also Q*(*x*_*i*_|*y*; *P*(*X*)) using logistic regression. Precisely,

$$\begin{array}{c} Q^{*}\left(x_{i}|y;P\left(X\right)\right)\approx {\hat{P}}_{lr}\left(x_{i}|Y=y;P\left(X\right)\right), \end{array}$$ (25)

where ${\hat{P}}_{lr}\left(x_{i}|Y=y;P\left(X\right)\right)$ denotes the logistic regression model, Eq 10, for classifying *x*_*i*_ based on responses, *y*, and the input distribution, *P*(*X*). Moreover, in Lemma 6 in S1 Text we show that the parameters of logistic regression have to be estimated only once. Precisely, change of *P*(*X*) from *P*^(*k*−1)^(*X*) to *P*^(*k*)^(*X*) at each step of the iterative procedure requires only an update of the intercept parameters *α*_*i*_, for *i* from 1 to *m* − 1, according to the following formula

$$\begin{array}{c} \alpha_{i}^{\left(k\right)}=\alpha_{i}^{\left(k-1\right)}+\text{log}\;(\frac{P^{\left(k-1\right)}\left(x_{m}\right)}{P^{\left(k-1\right)}\left(x_{i}\right)})-\text{log}\;(\frac{P^{\left(k\right)}\left(x_{m}\right)}{P^{\left(k\right)}\left(x_{i}\right)}), \end{array}$$ (26)

where $\alpha_{i}^{\left(k\right)}$ is the intercept parameter of the logistic regression model, Eq 10, at *k*-th iteration of the alternate maximization procedure. Parameters, *β*_*i*_, for *i* = 1 to *m* − 1, remain the same for all iterations.

#### Pseudo-code

The above five elements are combined into the algorithm to compute information capacity, C* in the following way. Primarily, the initial values of the input distribution, denoted as *P*^(0)^(*X*), are set, e.g., to be equal to relative frequencies of measurements available for each input, $P^{\left(0\right)}\left(x_{i}\right)=\frac{n_{i}}{\sum_{r=1}^{m}n_{r}}$. Thereafter, logistic regression model is constructed to obtain initial value of the function *Q*(), denoted as *Q*^(0)^, i.e., $Q^{\left(0\right)}\left(x_{i}|y;P^{\left(0\right)}\left(X\right)\right)={\hat{P}}_{lr}\left(x_{i}|Y=y;P^{\left(0\right)}\left(X\right)\right)$. Thereafter, at each iteration, *k*, distribution *P*(*X*), and function *Q*(*X*|*Y*) are updated, as well as the approximation of the capacity provided by the function *J*() is evaluated according to Eq 12. Iterations are repeated until convergence is reached. The algorithm is summarized as a pseudo-code in Box 1.

**Box 1 Algorithm to calculate channel capacity using statistical learning**

1: Set maximum number of iterations MAXIT and tolerance level tol

2: Set *k* = 1

3: Initialise *C*^(*k*)^, e.g. *C*^(−1)^ = −∞, *C*^(0)^ = 0

4: Set initial distribution of *P*^(0)^(*X*): $P^{\left(0\right)}\left(x_{i}\right)=\frac{n_{i}}{\sum_{r=1}^{m}n_{r}}$

5: Estimate $Q^{\left(0\right)}\left(x_{i}|y;P^{\left(0\right)}\left(X\right)\right)={\hat{P}}_{lr}\left(x_{i}|Y=y;P^{\left(0\right)}\left(X\right)\right)$, i.e., by logistic regression model

6: **while** k ≤ MAXIT AND |*C*^(*k*−1)^ − *C*^(*k*−2)^| >tol **do**

7:  Calculate *D*_*i*_(*Q*^(*k*−1)^) by Monte Carlo integration

$$\begin{array}{c} D_{i}\left(Q^{\left(k-1\right)}\right)=E_{P(Y|X=x_{i})}({\text{log}}_{2}Q^{\left(k-1\right)}\left(x_{i}|y\right))\approx \frac{1}{n_{i}}\sum_{l=1}^{n_{i}}{\text{log}}_{2}Q^{\left(k-1\right)}\left(x_{i}|y_{l}^{i}\right), \end{array}$$

8:  Optimize max_*P*(*X*)_ *J*(*P*, *Q*^(*k*−1)^)

$$\begin{array}{c} P^{\left(k\right)}\left(x_{i}\right)=P^{*}\left(x_{i};Q^{\left(k-1\right)}\left(x_{i}|y\right)\right)=\frac{\text{exp}(D_{i}\left(Q^{\left(k-1\right)}\right))}{\sum_{r=1}^{m}\text{exp}\left(D_{r}\left(Q^{\left(k-1\right)}\right)\right)} \end{array}$$

9:  Optimize max_*Q*(*X*, *Y*)_ *J*(*P*^(*k*)^, *Q*) by

$$\begin{array}{c} Q^{\left(k\right)}\left(x_{i}|y\right)=Q^{*}\left(x_{i}|y;P^{\left(k\right)}\left(x_{i}\right)\right)={\hat{P}}_{lr}\left(x_{i}|Y=y;P^{\left(k\right)}\left(X\right)\right) \end{array}$$

which can be calculated from *Q*^(*k*−1)^(*x*_*i*_|*y*) according to Eq 26

10:  Get, *C*^(*k*)^, an estimate of C*

$$\begin{array}{c} C^{\left(k\right)}=\sum_{i=1}^{m}P^{\left(k\right)}\left(x_{i}\right)(D_{i}\left(Q^{\left(k\right)}\right)-\text{log}\;P^{\left(k\right)}\left(x_{i}\right)) \end{array}$$

11:  *k* = *k* + 1

12: **end while**

13: **return** C* = *C*^(*k*)^, P*(*X*) = *P*^(*k*)^(*X*).

### Practical calculation of the probabilities of correct discrimination

Probabilities of correct discrimination, PCDs defined in Eqs 6 and 7, are expressed in terms of probabilities *P*(*x*_*i*_|*Y* = *y*). Logistic regression model, Eq 10, on the other hand, provides estimates of these probabilities. Therefore, logistic regression estimates, ${\hat{P}}_{lr}\left(x_{i}|Y=y;P\left(X\right)\right)$, can be used to estimate probabilities of correct discrimination.

In order to estimate PCDs, for a given pair of input values *x*_*i*_ and *x*_*j*_, the logistic regression model needs to be fitted using response data corresponding to the two considered inputs, i.e. $y_{l}^{r}$, for *r* ∈ {*i*, *j*} and *l* ranging from 1 to *n*_*r*_. To ensure that both inputs have equal contribution to the calculated discriminability, equal probabilities should be assigned, *P*(*X*) = (*P*(*x*_*i*_), *P*(*x*_*j*_)) = (1/2, 1/2). Once the regression model is fitted, probability of assigning a given cellular response, *y*, to the correct input value is estimated as

$$\begin{array}{c} \text{max}\{{\hat{P}}_{lr}\left(x_{i}|Y=y;P\left(X\right)\right),{\hat{P}}_{lr}\left(x_{j}|Y=y;P\left(X\right)\right)\}. \end{array}$$

Note that *P*(*x*_*j*_|*Y* = *y*) = 1 − *P*(*x*_*i*_|*Y* = *y*) as well as ${\hat{P}}_{lr}\left(x_{j}|Y=y;P\left(X\right)\right)=1-{\hat{P}}_{lr}\left(x_{i}|Y=y;P\left(X\right)\right)$. Averaging the above over all responses corresponding to input values *x*_*i*_ and *x*_*j*_, i.e., with respect to the distribution $P\left(y\right)=\frac{1}{2}P\left(y|X=x_{i}\right)+\frac{1}{2}P\left(y|X=x_{j}\right)$, yields *PCD*(*x*_*i*_, *x*_*j*_)

$$\begin{array}{ccc} PCD(x_{i},x_{j}) & \approx & \frac{1}{2}\frac{1}{n_{i}}\sum_{l=1}^{n_{i}}\text{max}\left\{{\hat{P}}_{lr}\left(x_{i}|Y=y_{l}^{i};P\left(X\right)\right),{\hat{P}}_{lr}\left(x_{j}|Y=y_{l}^{i};P\left(X\right)\right)\right\}\\ & + & \frac{1}{2}\frac{1}{n_{j}}\sum_{l=1}^{n_{j}}\text{max}\left\{{\hat{P}}_{lr}\left(x_{i}|Y=y_{l}^{j};P\left(X\right)\right),{\hat{P}}_{lr}\left(x_{j}|Y=y_{l}^{j};P\left(X\right)\right)\right\}. \end{array}$$ (27)

To account for the possibility of overfitting of the regression model, the bootstrap procedure needs to be used [36]. For instance, available data should be randomly divided into a training data set, i.e., data set used to fit logistic regression, and test data set, i.e., the set to evaluate Eq 27. The average of the PCDs from the bootstrap procedure should be used as a final estimate of the probability of correct discrimination.

### Numerical validation

In order to validate the accuracy of the proposed information capacity estimators, examine the computational performance of the algorithm, and highlight advantages of the introduced approach, we have designed four test scenarios and carried out a comparison with the KNN method. We have chosen KNN for the comparison as it is virtually the only available technique that allows estimating *MI* and C* for systems with multidimensional output, *Y*. One of the test scenarios, Scenario 1, is presented below whereas the remaining three scenarios, Scenarios 2-4, are part of S1 Text. Scenario 1 demonstrates that the proposed approach, here further referred to as SLEMI, (i) provides more accurate estimates than the KNN method; (ii) provides robust, parameter-free estimates; and (iii) has desired properties in terms of the computational cost. Scenario 2 replicates diagnostics proposed for the KNN methods in reference [19] and demonstrates that, in contrast to the KNN method, accuracy of SLEMI estimates persists for high dimensionality of output data. Scenario 3 validates the accuracy of SLEMI estimates against several different shapes of the output distribution. Finally, Scenario 4 uses a model of a transcription factor activity [20] to demonstrate that SLEMI can be used to quantify the information capacity of frequency encoded signals.

**Fig 3:**
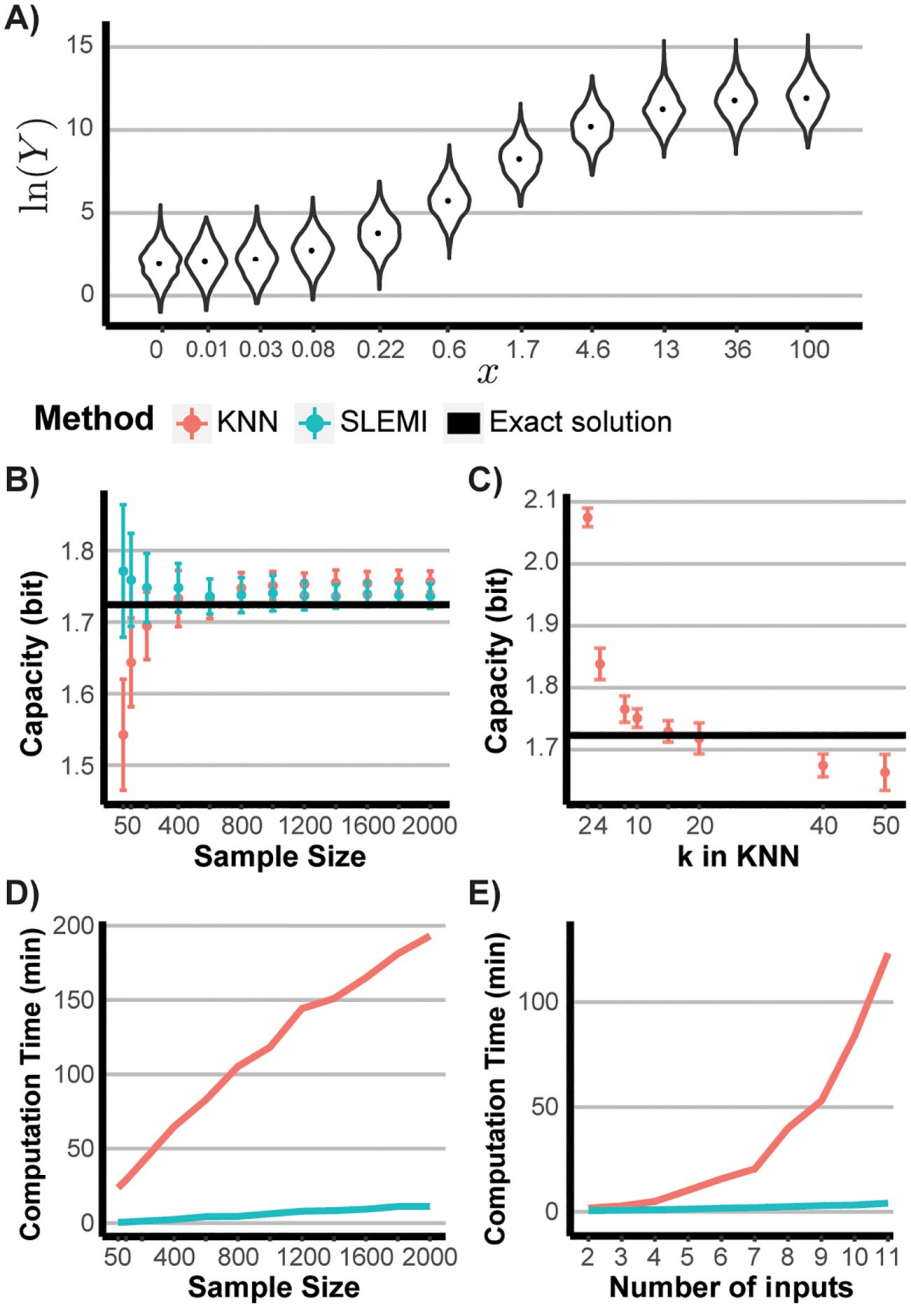
Test scenario 1. **(A)** A violin plot representation of the conditional output distribution *Y*|*x*_*i*_ for 11 considered inputs. **(B)** Information capacity estimates as the function of the sample size *N*. Blue and red lines correspond to SLEMI and KNN estimates, respectively. The bold black line marks the true value of the capacity. For the KNN estimation, *k* = 10 was assumed. **(C)** Information capacity estimates of the KNN method as a function of *k* compared with the true value (bold black line). The error-bars in B and C show the standard deviation of capacity estimates from 40 repeated samplings. *N* = 1000 was assumed. **(D)** Computation time of SLEMI and KNN method as the function of the sample size *N*. **(E)** Computation time of SLEMI (blue) and the KNN method as the function of the number of considered input values. Input values were subsequently added starting with *x*_1_ and *x*_2_, only, and ending up with all 11 considered input values. The times reported in panels (D) and (E) correspond to computations performed by a single core on a workstation with Intel Xeon E5-1650 3.50 GHz processor and 32 GB RAM.

The test model used as Scenario 1 aims to reflect a simple experimental setup in which one-dimensional responses of individual cells to a range of stimuli are quantified. Precisely, the test model considers a channel with the log-normally distributed output, *Y*. The mean, *μ*(*x*) and variance *σ*^2^ of the log-output are assumed to be the sigmoid function, and a constant, respectively. Precisely,

$$\begin{array}{c} Y|x_{i}∼\text{exp}\;(N(\mu \left(x\right),\sigma^{2})), \end{array}$$

for $\mu \left(x\right)=\frac{V\cdot x}{1+x}$, *V* = 10, *σ*^2^ = 1. For the above model, we considered eleven input values, *m* = 11. Input vales range from 0 to 100, *x*_*i*_ ∈ [0, 100]. Sample distributions of output corresponding to considered input values are shown in Fig 3A.

To test the estimation accuracy, we computed information capacity estimates using SLEMI and KNN method for different sample size, *N*, i.e., the number of data points corresponding to each input value used for estimation. True information capacity was evaluated numerically. Fig 3B presents both information capacity estimates, as well as the true value, as the function of *N*, for *N* ranging from 50 up to 2000. For sample sizes typical for biological experiments, i.e., tens or hundreds of measured cells, SLEMI clearly provides more accurate estimates than KNN.

In contrast to SLEMI, KNN estimates depend on the choice of the parameter *k*, whereas clear rules how *k* should be selected are missing [56–58]. For computation of the KNN estimates in Fig 3A we used *k* = 10, i.e., 10 closest points to each observation *y* were used to approximate the density *P*(*y*|*X* = *x*_*i*_). To highlight the impact of the parameter *k*, we re-calculated the KNN estimates of Fig 3B using *k* ranging from 2 to 50, at fixed *N* = 1000. As show in Fig 3C, selection of *k* has an impact on the value of the capacity estimates that may lead to considerable bias. SLEMI estimates are free from this disadvantage.

Further, we have examined how the computational time needed to obtain estimates scales with sample size, *N*, and the number of input values, *m*. Fig 3D presents computational times as a function on *N*. Both methods exhibit linear increase with *N* (Fig 3D). Computational time of SLEMI increases at the lower rate. Although we optimized implementations of both methods to ensure a fair comparison, the rate of increase may depend on specifics of the code used. Finally, we calculated the computational time as a function of the number of input values. Initially, we considered two input values, *m* = 2, and increased, one by one, up to eleven input values, *m* = 11. The computational time of SLEMI increases linearly, whereas computational time corresponding to KNN method increases at least quadratically (Fig 3E). Linear scaling with respect to the number of input values is important for the method to be applicable to study complex systems with multiple inputs.

In summary, the above test scenario shows that SLEMI provides more accurate estimates of the information capacity, C*, than the KNN method, especially for small sample size. Importantly, estimates are parameter-free, which contributes to robust estimation. Also, computational time scales linearly with respect to the number of input values. The test scenario presented above considered one-dimensional output, *Y*, as the demonstration of key benefits resulting from using SLEMI did not require a multidimensional complex model. The presented advantages, however, are of particular importance when systems with multivariate output, *Y*, are studied. For multivariate systems robust estimation of the information capacity, C*, with KNN method can be problematic due to the choice of the parameter *k* and numerical optimization. Therefore, in the test Scenario 2, in S1 Text, we have confirmed that, unlike for the KNN method, high accuracy of information capacity estimates persist for multidimensional data.

## Supporting information

S1 Text Supplementary information PDF file contains expanded description of theoretical and experimental methods. (PDF)
