# Supplementary material for: Information-theoretic analysis of multivariate single-cell signaling responses
Source: PLoS Comput Biol. 2019 Jul 12;15(7):e1007132. doi: 10.1371/journal.pcbi.1007132 (PMC6655862; doi:10.1371/journal.pcbi.1007132)
Supplement: S1 Text — (PDF) [file pcbi.1007132.s001.pdf]

Information-theoretic analysis of multivariate single - cell signaling responses

Supplementary Information

Tomasz Jetka[1], Tomasz Winarski[1], Karol Nienałtowski[1], Sławomir Błoński, & Michał Komorowski[1]

[1]Institute of Fundamental Technological Research,
Polish Academy of Sciences,
Warsaw, Poland

Contents

1 Background methods **3**
- 1.1 Mutual information . . . 3
- 1.2 Existing methods to estimate mutual information and information capacity for single - cell signaling data . . . 3

2 Maximisation algorithm to compute capacity **5**
- 2.1 Information capacity as the double maximization problem . . . 5
- 2.2 Individual maxima can be found explicitly . . . 6
- 2.3 Alternate maximisation . . . 7
- 2.4 Incorporation of logistic regression . . . 9

3 Numerical validation **12**
- 3.1 Test scenario 2: SLEMI ensures accurate estimation regardless of the dimension of the output, Y. . . 12
- 3.2 Test scenario 3: SLEMI provides accurate estimates regardless of the specific form of the output distribution . . . 14
- 3.3 Test scenario 4: toy model of frequency-encoded signals . . . 16

4 Analysis of the Nf-κB responses to TNF-α **18**
- 4.1 Experimental methods . . . 18
- 4.2 Image analysis . . . 18
- 4.3 Experimental data set . . . 20
- 4.4 Quantification of information capacity . . . 20
- 4.5 Redundant information in signaling dynamics . . . 21

1 Background methods

1.1 Mutual information

The Eq. 2 of the main paper provides one of the several equivalent definitions of the mutual information. The specific choice of the definition was motived by the need for concise presentation of the algorithm. However, the more intuitive definition of the mutual information, in the context of signaling systems, is based on entropy. Precisely, consider the input distribution $P(X) = (P(x_1), \dots, P(x_m))$. The distribution can be interpreted as the frequency at which different values occur. Therefore, the uncertainty of X associated with this distribution represents maximal uncertainty regarding the input signal. Within information theory, the uncertainty of the random variable can be quantified as entropy

$$H(X) = -\sum_{i=1}^{m} \log_2(P(x_i))P(x_i). \tag{1}$$

Entropy is expressed in bits and, simplistically, quantifies the uncertainty of the phenomenon represented by X. The less predictable is the value of X, the larger is the entropy. For example, when X is defined on a finite set, the largest entropy is manifested by a uniform distribution, in the case of which all possible outcomes are equally likely to occur and therefore there is no information which value of X to expect. Observation of the output has a potential to reduce uncertainty regarding the input value. Via the Bayes formula, plausible inputs that generated a specific output value, y, are represented as the probability distribution

$$P(X|Y = y) = \frac{P(Y = y|X)P(X)}{P(Y = y)}. \tag{2}$$

Uncertainty regarding input value can be then quantified by the entropy of the distribution $P(X|Y = y)$

$$H(X|Y = y) = -\sum_{i=1}^{m} \log_2(P(x_i|Y = y))P(x_i|Y = y)dx. \tag{3}$$

As the output is random, averaging $H(X|Y = y)$ over all possible outputs quantifies the average uncertainty regarding the input, given the output, $H(X|Y)$,

$$H(X|Y) = \int_{\mathcal{Y}} H(X|Y = y)P(y)dy, \tag{4}$$

where $\mathcal{Y}$ is the space of possible values of the output, Y, and

$$P(y) = \sum_{i=1}^{m} P(y|X = x_i)P(x_i) \tag{5}$$

is the marginal distribution of the output. The difference between $H(X)$ and $H(X|Y)$ is equal to the mutual information, Eq. 2 of the main paper,

$$MI(X,Y) = H(X) - H(X|Y). \tag{6}$$

The above demonstrates that MI measures the average reduction in uncertainty, expressed in bits, regarding the input resulting from observing the output.

1.2 Existing methods to estimate mutual information and information capacity for single - cell signaling data

The conventional solution to the problem of estimating the channel capacity, Eq. 3 of the main paper, is provided by the Blahut-Arimoto (BA) algorithm [2, 3] and its extensions [24]. In its original version BA algorithm can be used to calculate the information capacity for a scenario, in which both the output, Y,

and the input, x, take discrete values, i.e. a finite set of values. It can also be applied to systems with continuous outputs, through output discretization, i.e., conversion into a variable that takes finite set values. A systematic way to convert the output into a discreet variable within the BA framework was proposed in [5]. The discretization of the output is an efficient solution only if Y is one-dimensional. Therefore, BA algorithm cannot efficiently estimate the capacity in scenarios with continuous multidimensional output, Y.

A method to calculate the capacity for signaling systems with continuous output of several dimensions have been recently proposed in [17], similarly to the work of [23] and earlier theoretical approaches of [11]. The method involves approximation of the output probability densities $P(Y|x_i)$, for $i = 1, ..., m$ with the k-nearest neighbour approach (KNN) and numerical optimization. Broadly speaking, the KNN method utilizes the distance to the kth nearest neighbor to approximate the probability density. The method is however problematic as the estimates depend on the selection of k and requires a large sample size to provide accurate results. Moreover, it involves numerical gradient optimization that is susceptible to finding local maxima, especially if the number of input values is large.

A more technical argument against KNN based estimation of the information capacity can also be made. The method proposed in [17] uses KNN directly without additional bias correction (compare with [11]) and for that reason is prone to inaccurate estimation of the mutual information for small sample size and high dimensionality of data. As a result, accurate estimation of information capacity requires accurate estimation of density. KNN density estimators have been shown to work well for large data size, i.e., to be consistent, for k increasing with the data size. Precisely, it is required that $k = k_N \to \infty$ and $k_N/N \to 0$ as $N \to \infty$ [12], where N is the number of data points used in estimation. The rate of the estimators' convergence, i.e., the number of data points needed to obtain the correct value of the estimated density, depends on the dimension d, of the random variable, Y. Therefore, for larger d, larger N and k may be required to get accurate estimates of the density $P(Y|x_i)$ and consequently of the capacity, C^*. Moreover, the optimal k does not follow a simple scaling rule with d and N. In [1], authors suggest to choose k, that is proportional to $n^{4/(d+4)}$, however for lower dimensions such conditions lead to highly biased entropy estimators. A more detailed discussion of those difficulties in a specific case of information theoretic measures can be found in [7, 14, 18]. In summary, the above arguments suggest that the selection of k, which leads to an accurate estimation of the information capacity, may be problematic.

2 Maximisation algorithm to compute capacity

Below, we formulate and prove technical lemmas referred to in the description of the maximisation algorithm in the *Methods* section of the main paper. The Lemmas are largely modifications of results formulated in the insightful book [25]. These were modified to account for continuous and multivariate output, Y.

2.1 Information capacity as the double maximization problem

The lemma below demonstrates that the Theorem 1a of the [3] works for continuous and multidimensional outputs Y.

Lemma 1. *Assume a discreet strictly positive input probability distribution $P(X) = (P(x_1), ..., P(x_m))$, i.e. for each x_i, $P(x_i) > 0$, and the conditional output probability distribution $P(Y|X = x_i)$, where Y is a continuous, possibly multivariate random variable. Then, the information capacity, Eq. 3 of the main paper, can be written as*

$$C^* = \max_{P(X)} MI(X,Y) = \max_{P(X)} \max_{Q(X|Y)} J(P(X), Q(X|Y)), \tag{7}$$

where $Q(X|Y)$ is a real-valued function that for a given $Y = y$ is a discrete probability distribution with respect to X such that

$$Q(x_i|y) = 0 \text{ if and only if } P(y|X = x_i) = 0, \tag{8}$$

whereas the auxiliary function J is defined as

$$J(P(X), Q(X|Y)) = \sum_{i=1}^{m} P(x_i) \int_{\mathcal{Y}} P(y|X = x_i) \log_2 \frac{Q(x_i|y)}{P(x_i)} dy \tag{9}$$

where $\mathcal{Y} \subset \mathbb{R}^d$ such that for all $y \in \mathcal{Y}$, $P(y|X = x_i) > 0$ for some x_i.

Proof It is enough to prove that

$$MI(X,Y) = \max_{Q(X|Y)} J(P(X), Q(X|Y)) = \max_{Q(X|Y)} \sum_{i=1}^{m} P(x_i) \int_{\mathcal{Y}} P(y|X = x_i) \log_2 \frac{Q(x_i|y)}{P(x_i)} dy.$$

Let's define

$$Q^*(x_i|y) = \frac{P(x_i)P(y|X = x_i)}{\sum_{r=1}^{m} P(x_r)P(y|X = x_r)}.$$

Notice, that $Q^*(x_i|y)$ is well defined under assumtions of Eq. 9. Then,

$$J(P(X), Q^*(X|Y)) = \sum_{i=1}^{m} P(x_i) \int_{\mathcal{Y}} P(y|X = x_i) \log_2 \frac{Q^*(x_i|y)}{P(x_i)} dy =$$

$$= \sum_{i=1}^{m} P(x_i) \int_{\mathcal{Y}} P(y|X = x_i) \log_2 \left(\frac{P(x_i)P(y|X = x_i)}{\sum_{r=1}^{m} P(x_r)P(y|X = x_r)} \frac{1}{P(x_i)} \right) dy =$$

$$= \sum_{i=1}^{m} P(x_i) \int_{\mathcal{Y}} P(y|X = x_i) \log_2 \left(\frac{P(y|X = x_i)}{\sum_{r=1}^{m} P(x_r)P(y|X = x_r)} \right) dy =$$

$$= \sum_{i=1}^{m} P(x_i) \int_{\mathcal{Y}} P(y|X = x_i) \log_2 \frac{P(y|X = x_i)}{P(y)} dy = MI(X,Y),$$

which results in

$$J(P(X), Q^*(X|Y)) = MI(X, Y).$$

We introduce

$$q(y) = \sum_{r=1}^{m} P(x_r) P(y|X = x_r).$$

Then, we calculate the difference

$$MI(X,Y) - J(P(X), Q(X|Y)) = J(P(X), Q^*(X|Y)) - J(P(X), Q(X|Y)) =$$

$$= \sum_{i=1}^{m} P(x_i) \int_{\mathcal{Y}} P(y|X = x_i) \log_2 \frac{Q^*(x_i|y)}{P(x_i)} dy - \sum_{i=1}^{m} P(x_i) \int_{\mathcal{Y}} P(y|X = x_i) \log_2 \frac{Q(x_i|y)}{P(x_i)} dy =$$

$$= \sum_{i=1}^{m} \int_{\mathcal{Y}} q(y) Q^*(x_i|y) \log_2 \frac{Q^*(x_i|y)}{Q(x_i|y)} dy \overset{(*)}{\geq} \frac{1}{\log 2} \left(\sum_{i=1}^{m} \int_{\mathcal{Y}} q(y) Q^*(x_i|y) dy - \sum_{i=1}^{m} \int_{\mathcal{Y}} q(y) Q(x_i|y) dy \right) =$$

$$= \frac{1}{\log 2} \left(\int_{\mathcal{Y}} q(y) \sum_{i=1}^{m} Q^*(x_i|y) dy - \int_{\mathcal{Y}} q(y) \sum_{i=1}^{m} Q(x_i|y) dy \right) =$$

$$= \frac{1}{\log 2} \left(\int_{\mathcal{Y}} q(y) \left(\underbrace{\sum_{i=1}^{m} Q^*(x_i|y)}_{=1} - \underbrace{\sum_{i=1}^{m} Q(x_i|y)}_{=1} \right) dy \right) = 0,$$

where the inequality $(*)$ is based on a basic property of a logarithmic function

$$\log_2 x \geq \frac{1}{\log 2} \left(1 - \frac{1}{x} \right) \text{ with equality iff } x = 1.$$

In consequence, we proved that $MI(X,Y) = J(P(X), Q^*(X|Y))$ and $MI(X,Y) - J(P(X), Q(X|Y)) \geq 0$ with the equality, when $Q(X|Y) = Q^*(X|Y)$, which concludes the lemma.

2.2 Individual maxima can be found explicitly

The lemma below demonstrates that the Theorem 1b and 1c of the [3] works for continuous and multidimensional outputs Y and provides an explicit solution of the maximisation

$$P^*(X; Q(X,Y)) = \arg\max_{P(X)} J(P(X), Q(X|Y)).$$

Lemma 2. *The optimization problem*

$$\max_{P(X)} J(P(X), Q(X,Y)) \tag{10}$$

$$w.r.t. \sum_{i=1}^{m} P(x_i) = 1,$$
$$0 \leq P(x_i) \leq 1 \text{ for each } i$$

with $Q(x_i|y)$ being a probability function for each y is solved by

$$P^*(x_i) = \frac{\exp(D_i \log 2)}{\sum_{r=1}^{m} \exp(D_r \log 2)},$$

where $D_i = \int_{\mathcal{Y}} P(y|X = x_i) \log_2 Q(x_i|y) dy$ and $P(y|X = x_i)$ being a probability density function for each x_i.

Proof Let's reformulate the objective function

$$\max_{P(X)} J(P(X), Q(X|Y)) = \sum_{i=1}^{m} \int_{\mathcal{Y}} P(x_i)P(y|X=x_i) \log_2 \frac{Q(x_i|y)}{P(x_i)} dy =$$

$$= \sum_{i=1}^{m} \int_{\mathcal{Y}} P(x_i)P(y|X=x_i) \log_2 Q(x_i|y)dy - \sum_{i=1}^{m} \int_{\mathcal{Y}} P(x_i)P(y|X=x_i) \log_2 P(x_i)dy =$$

$$= \sum_{i=1}^{m} P(x_i) \int_{\mathcal{Y}} P(y|X=x_i) \log_2 Q(x_i|y)dy - \sum_{i=1}^{m} P(x_i) \log_2 P(x_i) \int_{\mathcal{Y}} P(y|X=x_i)dy =$$

$$= \sum_{i=1}^{m} P(x_i)D_i - \sum_{i=1}^{m} P(x_i) \log_2 P(x_i) = \sum_{i=1}^{m} P(x_i) \left(D_i - \log_2 P(x_i)\right),$$

where $D_i = \int_{\mathcal{Y}} P(y|X=x_i) \log_2 Q(x_i|y)dy$ does not depend on $P(x_i)$ and can be obtained by sample averaging . Therefore the initial optimization problem can be reformulated as

$$\max_{P(X)} \quad \sum_{i=1}^{m} P(x_i) \left(D_i - \log_2 P(x_i)\right) \tag{11}$$

$$\text{w.r.t.} \quad \sum_{i=1}^{m} P(x_i) = 1 \tag{12}$$

$$0 \le P(x_i) \le 1 \text{ for each } i \tag{13}$$

which can be easily solved by Lagrange multipliers method that yields system of equations

$$\begin{aligned}
0 &= D_1 - \log_2 P(x_1) - 1 - \lambda \quad \Rightarrow \quad \lambda = D_1 - \log_2 P(x_1) - 1, \\
&\vdots \qquad\qquad\qquad\qquad\qquad\qquad\quad \vdots \\
0 &= D_m - \log_2 P(x_m) - 1 - \lambda \quad \Rightarrow \quad \lambda = D_m - \log_2 P(x_m) - 1, \\
0 &= \left(\sum_{i=1}^{m} P(x_i)\right) - 1.
\end{aligned}$$

It follows that for any i

$$\frac{D_i - \log_2 P(x_i) - 1}{D_1 - \log_2 P(x_1) - 1} = 1 \Rightarrow \frac{P(x_i)}{P(x_1)} = \frac{\exp\left(D_i \log 2\right)}{\exp\left(D_1 \log 2\right)}$$

and using the fact that $\sum_{i=1}^{m} P(x_i) = 1$, we arrive at the formula

$$P^*(x_i) = \frac{\exp(D_i \log 2)}{\sum_{r=1}^{m} \exp(D_r \log 2)},$$

what was to prove.

2.3 Alternate maximisation

Below, we demonstrate convergence of the alternate maximisation (AM), defined by Eq. 19 and 20 of the main paper. This is done in two steps. First, in Lemma 3 we recall a general result for optimization of concave functions [25] (Section 9.1). In the subsequent Lemma 4, the Lemma 3 is applied to the function $J(P(X), Q(X|Y))$, Eq. 12 of the main paper, which proves the AM convergence. Lemma 4, together with its prove, is analogous to Lemma 9.1 and Theorem 9.2 in [25], where it was introduced for discrete output, Y.

Lemma 3. *Let $f(u_1, u_2)$ be a real-valued function and consider the optimization problem*

$$f^* = \max_{u_1 \in \mathcal{U}_1, u_2 \in \mathcal{U}_2} f(u_1, u_2).$$

In addition, denote by $u_2^*(u_1)$ the solution of $\arg\max_{u_2 \in \mathcal{U}_2} f(u_1, u_2)$ and by $u_1^*(u_2)$ the solution of $\arg\max_{u_1 \in \mathcal{U}_1} f(u_1, u_2)$. A sequence $\left(u_1^k, u_2^k\right)$ is constructed as follows:

1. *(u_1^0, u_2^0) is an arbitrary starting point from set $\mathcal{U}_1 \times \mathcal{U}_2$*
2. *for $k > 0$*

$$\begin{cases} u_1^k &= u_1^*\left(u_2^{k\text{-}1}\right) \quad \text{for } k > 0. \\ u_2^k &= u_2^*\left(u_1^k\right), \quad \text{for } k > 0. \end{cases}$$

Then, the sequence $\left(u_1^k, u_2^k\right)$ converges to the optimal solution, i.e. $f(u_1^k, u_2^k) \xrightarrow{k\to\infty} f^*$, if

- *function f is bounded from above,*
- *function f is continuous and has continuous partial derivatices,*
- *function f is concave,*
- *sets $\mathcal{U}_1$ and $\mathcal{U}_2$ are convex.*

The proof of the Lemma 3 is given in [25] (Section 9.1), while the use of AM in the case of mutual information and channel capacity is justified by [2, 3] and summarised in the next lemma.

Lemma 4. *Assume arbitrary distributions $P^{(0)}(x_i), Q^{(0)}(x_i|y)$, which correspond to $P(X)$ and $Q(X|Y)$, respectively. At each step, indexed by k, new $P(X)$ and $Q(X|Y)$ denoted as $P^{(k)}(x_i), Q^{(k)}(x_i|y)$, are defined by using the solutions of the individual maximization problems based on the solutions of the previous step*

$$\begin{aligned} P^{(k)}(x_i) &= P^*(x_i; Q^{(k-1)}(x_i|y)) \quad \text{for } k > 0, \\ Q^{(k)}(x_i|y) &= Q^*(x_i|y; P^{(k)}(x_i)) \quad \text{for } k > 0. \end{aligned} \tag{14}$$

Then, the above iterative scheme converges to the solution of the joint maximisation problem, Eq. 7, precisely

$$P^{(k)}(x_i) \xrightarrow{k\to\infty} P^*(x_i)$$

and

$$J(P^{(k)}(X), Q^{(k)}(X|Y)) \xrightarrow{k\to\infty} C^*,$$

where

$$C^* = \max_{P(X) \in \mathcal{U}_1} \max_{Q(X|Y) \in \mathcal{U}_2} J(P(X), Q(X|Y))$$

and

$$\mathcal{U}_1 = \left\{(P(x), x \in \mathcal{X}) : P(x) > 0, \textstyle\sum_{i=1}^m P(x_i) = 1\right\},$$

$$\begin{aligned} \mathcal{U}_2 = \Big\{ & \left(Q(x|y),\ x \in \mathcal{X},\ y \in \mathbb{R}^d\right) : \\ & Q(x|y) > 0 \text{ if } P(X = x_i|y) > 0, \\ & Q(x|y) = 0 \text{ if } P(y|X = x) = 0 \text{ and} \\ & \textstyle\sum_{i=1}^m Q(x_i|y) = 1 \text{ for all } y \in \mathbb{R}^d \Big\}. \end{aligned}$$

Proof

By Lemma 3 applied to $f(u_1, u_2) = J(P, Q)$, it is enough to prove that
(i) function J is bounded from above;
(ii) function J is continuous and its partial derivatives are continuous;
(iii) function J is concave;

(iv) sets $\mathcal{U}_1$,$\mathcal{U}_2$ are convex.

(i) $J(P,Q)$ is bounded, as by Lemma 1 and basic properties of mutual information and entropy [6] we have

$$J(P,Q) \leq MI(X,Y) \leq H(X) \leq \log_2(|\mathcal{X}|) = \log_2(m).$$

(ii) An argument for the continuity of function $J(P,Q)$ and its derivatives is given in [25]. Similarly, in our case, all the probabilities involved in the expression are strictly positive. In addition, differentiation of the integral is valid due to Lebesgue's dominated convergence theorem, since the whole expression is bounded (previous condition).

(iii) Now, we show that $J(P,Q)$ is a concave function. Consider (P_1,Q_1), $(P_2,Q_2) \in \mathcal{U}_1 \times \mathcal{U}_2$ and $\lambda \in [0,1]$, $\tilde{\lambda} = 1-\lambda$. Then, by log-sum inequality ([25], Theorem 2.32), we can write

$$\left(\lambda P_1(x) + \tilde{\lambda} P_2(x)\right) \log_2 \frac{\lambda P_1(x) + \tilde{\lambda} P_2(x)}{\lambda Q_1(x|y) + \tilde{\lambda} Q_2(x|y)} \leq \lambda P_1(x) \log_2 \frac{P_1(x)}{Q_1(x|y)} + \tilde{\lambda} P_2(x) \log_2 \frac{P_2(x)}{Q_2(x|y)}.$$

Inverting fractions in the logarithms

$$\left(\lambda P_1(x) + \tilde{\lambda} P_2(x)\right) \log_2 \frac{\lambda Q_1(x|y) + \tilde{\lambda} Q_2(x|y)}{\lambda P_1(x) + \tilde{\lambda} P_2(x)} \geq \lambda P_1(x) \log_2 \frac{Q_1(x|y)}{P_1(x)} + \tilde{\lambda} P_2(x) \log_2 \frac{Q_2(x|y)}{P_2(x)}.$$

By multiplying the above by $P(y|X=x_i)$, summing over all x and integrating over all y, we obtain

$$\sum_{i=1}^{m} \int_{\mathcal{Y}} P(y|X=x_i) \left(\lambda P_1(x_i) + \tilde{\lambda} P_2(x_i)\right) \log_2 \frac{\lambda Q_1(x_i|y) + \tilde{\lambda} Q_2(x_i|y)}{\lambda P_1(x_i) + \tilde{\lambda} P_2(x_i)} \geq$$

$$\geq \sum_{i=1}^{m} \int_{\mathcal{Y}} \lambda P(y|X=x_i) P_1(x_i) \log_2 \frac{Q_1(x_i|y)}{P_1(x_i)} + \sum_{i=1}^{m} \int_{\mathcal{Y}} \tilde{\lambda} P(y|X=x_i) P_2(x_i) \log_2 \frac{Q_2(x_i|y)}{P_2(x_i)}.$$

It can be rewritten as

$$J(\lambda P_1 + \tilde{\lambda} P_2, \lambda Q_1 + \tilde{\lambda} Q_2) \geq \lambda J(P_1,Q_1) + \tilde{\lambda} J(P_2,Q_2),$$

which proves that $J(P,Q)$ is concave.

(iv) It is straightforward to check that subsets $\mathcal{U}_1$,$\mathcal{U}_2$ are convex. For example, let $P_1, P_2 \in \mathcal{U}_1$. Then, for $\lambda \in (0,1)$, $\tilde{P} = \lambda P_1 + (1-\lambda) P_2 > 0 + 0 = 0$ and

$$\sum_x \tilde{P}(x) = \sum_x \lambda P_1(x) + \sum_x ((1-\lambda) P_2(x)) = \lambda \cdot 1 + (1-\lambda) \cdot 1 = 1.$$

Therefore, $\tilde{P} \in \mathcal{U}_1$. Analogous argument applies to the set $\mathcal{U}_2$.

Eventually, conditions (i-iv) are proven and by Lemma 3, iterative scheme from Eq.14 converges, $P^k \to P^*$ and $J(P^k, Q^k) \to C^*$.

2.4 Incorporation of logistic regression

The optimization algorithm assumes that in each iteration, $P^{(k)}(x_i)$ and $Q^{(k)}(x_i|y)$ are calculated based on $P^{(k-1)}(x_i)$ and $Q^{(k-1)}(x_i|y)$. Given that $Q^{(k)}(x_i|y) \approx \hat{P}_{lr}(x_i|Y=y; P^{(k)}(X))$, it means that in each step of the iteration, a full estimation of logistic regression model needs to be performed. However, in practice $Q^{(k)}(x_i|y)$ in step k can be analytically calculated from $Q^{(k-1)}(x_i|y)$. Lemmas 5 and 6 derive the update formula for the logistic regression parameters.

Lemma 5. *Let X be a discrete random variable with values in the set $\{x_1, \ldots, x_m\}$, while $P^I(x_i)$ and $P^{II}(x_i)$ are two different probability distributions defined over this set. Additionally, assume that distribution $P(Y|X = x_i)$ is fixed for each x_i and $\hat{P}^I_{lr}(X|Y = y)$ is the logistic regression model obtained from $P(Y|X)$ and $P^I(x_i)$. Then, the relationship for another logistic model, $\hat{P}^{II}_{lr}(X|Y = y)$, based on $P(Y|X)$ and $P^{II}(x_i)$ is given by formulas*

$$\frac{\hat{P}^{II}_{lr}(x_i|Y = y)}{\hat{P}^{II}_{lr}(x_m|Y = y)} \approx \frac{\hat{P}^{I}_{lr}(x_i|Y = y)}{\hat{P}^{I}_{lr}(x_m|Y = y)} \cdot \frac{P^I(x_m)}{P^I(x_i)} \cdot \frac{P^{II}(x_i)}{P^{II}(x_m)} \quad \textit{for each } i. \tag{15}$$

<u>*Proof*</u>

Logistic regression is used to predict labels of X from measurements of Y. Then, with the assumptions from paragraph *Logistic regression* in section Methods of the main paper, multinomial logistic regression estimates following equations

$$\hat{P}^I_{lr}(x_1|Y = y) = \frac{\exp\left(\alpha_1 + \beta_1^T y\right)}{1 + \sum_{r=1}^{m-1} \exp\left(\alpha_r + \beta_r^T y\right)},$$

$$\vdots$$

$$\hat{P}^I_{lr}(x_i|Y = y) = \frac{\exp\left(\alpha_i + \beta_i^T y\right)}{1 + \sum_{r=1}^{m-1} \exp\left(\alpha_r + \beta_r^T y\right)},$$

$$\vdots$$

$$\hat{P}^I_{lr}(x_{m-1}|Y = y) = \frac{\exp\left(\alpha_{m-1} + \beta_{m-1}^T y\right)}{1 + \sum_{r=1}^{m-1} \exp\left(\alpha_r + \beta_r^T y\right)},$$

$$\hat{P}^I_{lr}(x_m|Y = y) = \frac{1}{1 + \sum_{r=1}^{m-1} \exp\left(\alpha_r + \beta_r^T y\right)},$$

where $y \in \mathbb{R}^d$ is an observed vector of measurements, $\alpha_i \in \mathbb{R}$ are intercepts for i-th class and $\beta_i \in \mathbb{R}^d$ are vector of parameters for i-th class.

The logistic regression model $\hat{P}_{lr}(x_i|Y = y)$ is meant to approximate the true distribution $P(x_i|Y = y)$. On the other hand, using the Bayes formula and definitions of conditional probabilities

$$\frac{P(x_i|Y = y)}{P(x_m|Y = y)} = \frac{\frac{P(y|X=x_i)P(x_i)}{P(y)}}{\frac{P(y|X=x_m)P(x_m)}{P(y)}} = \frac{P(y|X = x_i)}{P(y|X = x_m)} \cdot \frac{P(x_i)}{P(x_m)}$$

and hence

$$\frac{P(y|X = x_i)}{P(y|X = x_m)} = \frac{P(x_i|Y = y)}{P(x_m|Y = y)} \cdot \frac{P(x_m)}{P(x_i)}.$$

If the logistic regression model $\hat{P}_{lr}(x_i|Y = y; P(X))$ is a good approximation of the distribution $P(x_i|Y = y)$, then

$$\frac{P(y|X = x_i)}{P(y|X = x_m)} \approx \frac{\hat{P}_{lr}(x_i|Y = y; P(X))}{\hat{P}_{lr}(x_m|Y = y; P(X))} \cdot \frac{P(x_m)}{P(x_i)}. \tag{16}$$

Notice that in our scenario, the left-hand side of the last equation is assumed to be fixed. Therefore, any changes in priors of the right-hand side must be accounted for by changes in logistic regression estimates. Eventually, using Eq. 16 twice for distributions $P^I(x_i)$ and $P^{II}(x_i)$, respectively, we get

$$\frac{\hat{P}^I_{lr}(x_i|Y = y)}{\hat{P}^I_{lr}(x_m|Y = y)} \cdot \frac{P^I(x_m)}{P^I(x_i)} \approx \frac{P(y|X = x_i)}{P(y|X = x_m)} \approx \frac{\hat{P}^{II}(x_i|Y = y)}{\hat{P}^{II}(x_m|Y = y)} \cdot \frac{P^{II}(x_m)}{P^{II}(x_i)}. \tag{17}$$

In consequence,

$$\frac{\hat{P}^{II}_{lr}(x_i|y)}{\hat{P}^{II}_{lr}(x_m|y)} \approx \frac{\hat{P}^{I}_{lr}(x_i|y)}{\hat{P}^{I}_{lr}(x_m|y)} \cdot \frac{P^{I}(x_m)}{P^{I}(x_i)} \cdot \frac{P^{II}(x_i)}{P^{II}(x_m)},$$

which gives a straightforward formula for updating $\hat{P}_{lr}(x_i|y)$.
We can also express the relation (15) in more details, using parameters of the logistic model. Indeed, if we substitute formulas of logistic regression into Eq. 15 we get (for any i)

$$\exp\left(\alpha^{II}_i + \left(\beta^{II}_i\right)^T y\right) = \exp\left(\alpha^{I}_i + \left(\beta^{I}_i\right)^T y\right) \frac{\frac{P^I(x_m)}{P^I(x_i)}}{\frac{P^{II}(x_m)}{P^{II}(x_i)}}.$$

This implies that for each $i \in \{1, \dots, m\}$ and for any $k > 0$

$$\alpha^{II}_i = \alpha^{I}_i + \log\frac{P^I(x_m)}{P^{II}(x_m)} - \log\frac{P^I(x_i)}{P^{II}(x_i)},$$

$$\beta^{II}_i = \beta^{I}_i,$$

what means that only intercepts of the logistic regression model changes with the change of the underlying probabilities $P(X)$.

Lemma 6. *Consider $(k-1)$ and k-th steps of the algorithm presented in Box 1 in main paper. Then,*

$$Q^{(k-1)}(x_i|y) = \hat{P}_{lr}(x_i|Y = y; P^{(k-1)}(X))$$

is a logistic regression model estimated with the assumption that X follows the distribution $P^{(k-1)}(X)$. Let $P^{(k)}(x_i)$ be an updated input distribution. In order to complete the iteration step, i.e. to find parameters of

$$Q^{(k)}(x_i|y) = \hat{P}_{lr}(x_i|Y = y; P^{(k)}(X))$$

one can use the following formula

$$\frac{Q^{(k)}(x_i|y)}{Q^{(k)}(x_m|y)} \approx \frac{Q^{(k-1)}(x_i|y)}{Q^{(k-1)}(x_m|y)} \cdot \frac{P^{(k-1)}(x_m)}{P^{(k-1)}(x_i)} \cdot \frac{P^{(k)}(x_i)}{P^{(k)}(x_m)}.$$

which relates odds ratio of $Q^{(k-1)}(x_i|y)$ and $Q^{(k)}(x_i|y)$.
Proof
To obtain the above relation, for any $k > 0$, it is enough to use following substitutions in the Lemma 5: $P^I = P^{(k-1)}(x_i)$, $P^{II} = P^{(k)}(x_i)$ and $\hat{P}^I_{lr} = Q^{(k-1)}(x_i|y)$, $\hat{P}^{II}_{lr} = Q^{(k)}(x_i|y)$.
In addition, the consequence of the above is also the correspondence between parameters of logisitic regression model between each step:

$$\alpha^k_i = \alpha^{k-1}_i + \log\frac{P^{k-1}(x_m)}{P^{k-1}(x_i)} - \log\frac{P^k(x_m)}{P^k(x_i)},$$

$$\beta^k_i = \beta^{k-1}_i.$$

3 Numerical validation

Below, we present three test scenarios, which in addition to the test scenario 1 presented of the main paper, were used to numerically validate the method and to demonstrate its advantages.

3.1 Test scenario 2: SLEMI ensures accurate estimation regardless of the dimension of the output, Y.

As discussed in Section 1.2, the KNN-based method is virtually the only available method that enables computation of the information capacity for systems with multidimensional outputs, Y. To show that our method indeed provides a significant advantage over the KNN based estimation of information capacity, we replicate a test model introduced by the authors of KNN based method [17].
The test model considers a channel with two possible input values, $X \in \{x_1, x_2\}$, and output $Y|X = x_i$ given by a d-dimensional Gaussian distributions with identical, diagonal covariance matrices, and mean vectors that are the same except the first dimension. Precisely,

$$Y|x_i \sim \mathcal{N}(\mu_i, \Sigma); \quad Y \in \mathbb{R}^d, \text{ for } i = 1, 2;$$

$$\mu_1 = (0, 0, \ldots, 0); \quad \mu_2 = (2, 0, \ldots, 0);$$

$$\Sigma = \begin{pmatrix} 1 & 0 & \ldots & 0 \\ 0 & 1 & \ldots & 0 \\ \vdots & \vdots & \ddots & \vdots \\ 0 & 0 & \ldots & 1 \end{pmatrix}.$$

For the above model, we used our approach and the KNN based method to calculate capacities for different dimensions, d, of the output Y. Besides, we considered different sample sizes, N, i.e., the number of observations corresponding to each input value. The KNN based method requires specification of the parameter k that determines the number of neighboring data points that are used to estimate the probability density of the output. Following [17], we initially set $k = 10$, and, thereafter, analysed dependence of the estimates on k.

Fig. I.A shows capacities estimated using our approach (blue) and the KNN based method (red) for d ranging from 2 up to 30, for three sample sizes, N, 500, 2000, and 4000. For reference, true capacity calculated using numerical integration and optimization of the exact model is also plotted. Clearly, our approach outcompetes the KNN method in terms of bias, i.e. difference between the mean estimate and the true value, for all dimensions and sample sizes. The bias of the KNN based estimates is most striking for the smallest considered sample size ($N = 500$). Moreover, Fig IB shows that estimates obtained with our method converge quickly, with increasing N, to the true value of capacity, whereas estimates of the KNN method remain biased even for large sample size. Computation times corresponding to panels A and B are plotted in panels C and D, respectively. In this example, our method is approximately two-fold faster than the KNN approach. Relatively minor gain results from the model having only two input values. Further, in test scenario 2, we show that the gain can 100-fold for > 10 input values.

Importantly, in contrast to the KNN based method, our approach is robust to arbitrary assumptions regarding the parameters of the algorithm. Precisely, the KNN method involves setting the parameter k that determines the number of neighboring data points that are used to estimate the probability density of the output. As a result, the obtained capacity estimates are not robust. Our approach is free from such assumptions and, hence, guarantees robust estimation. For the KNN based estimates presented in Fig. I.A-B, we have used $k = 10$, as suggested by the authors of [17]. However, the estimate values are influenced by the choice of the parameter k. Therefore, the comparison of Fig. I.A-B present a scenario that is optimistic for the KNN based approach. In Fig. I.E, we show that the choice of k can introduce even stronger bias of the KNN based information capacity estimates, which further demonstrates the advantage of our method.

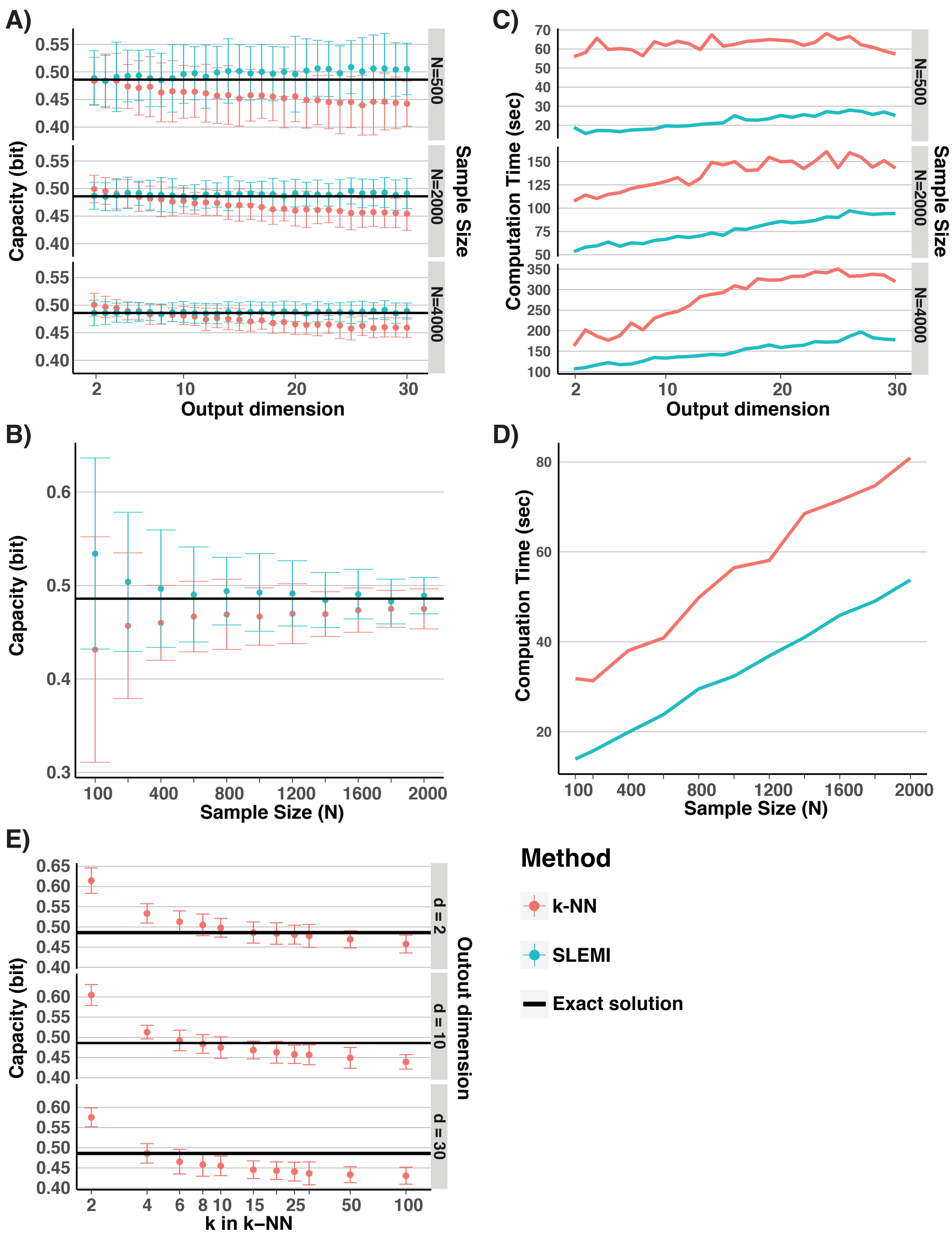

Figure I: Test scenario 2. **(A)** Information capacity estimates as a function of the dimension d for three different sample sizes N. Blue and red dots correspond to our method and the KNN based method, respectively. Black bold line marks the true value of capacity. **(B)** Information capacity estimates as a function of the sample size N for $d = 10$. Lines are coloured as in (A). **(C)** Computation time needed to obtain a single estimate in (A). **(D)** Computation time needed to obtain a single estimate in (B). **(E)** Information capacity estimates of the KNN based method as a function of k for three different dimension d and $N = 2000$. The error-bars in all panels (A),(B), and (E) show standard deviation of capacity estimates from 40 repeated samplings. In panels (A-D), k=10 was assumed. The times reported in panels (C) and (D) correspond to computations performed by a single core on a workstation with Intel® Xeon® E5-1650 3.50 GHz processor and 32 GB RAM.

3.2 Test scenario 3: SLEMI provides accurate estimates regardless of the specific form of the output distribution

The theory described in Section 2 guarantees the correctness of our approach if the classifier based on logistic regression, $\hat{P}_{lr}(x_i|y)$, is a good approximation of the conditional input probabilities, $P(x_i|y)$. The approximation may not be accurate if the conditional input probabilities cannot be represented by the functional form assumed by logistic regression, Eq. 9 of the main paper. The conditional input probabilities, $P(x_i|y)$, are dependent, via Bayes formula, on the conditional output probabilities, $P(y|x_i)$. Therefore, to test the robustness of our approach against different forms of the conditional input distributions, $P(x_i|y)$, we have assumed various output distributions, $P(y|x_i)$. Our test strategy is also implied by practical applications in which, input distributions, $P(x_i|y)$, are functions of experimentally measured output distributions, $P(y|x_i)$.
Specifically, we have examined four different types of univariate conditional output probability densities $P(y|x_i)$, with five possible input values, $X \in \{x_1, \dots, x_5\}$ (Fig. II.A). Precisely, we considered

Type 1: $Y|x_i \ \sim$ Exponential,

Type 2: $Y|x_i \ \sim$ Gamma,

Type 3: $Y|x_i \ \sim$ Normal,

Type 4: $Y|x_i \ \sim$ Lognormal.

For each of the above distributions, we have assumed that

a) the difference between expected values of subsequent output's conditional distributions is fixed at 1

$$\mathbb{E}(Y|X = x_{i+1}) - \mathbb{E}(Y|X = x_i) = 1,$$

b) for each i, samples of data are of the same size, N,

$$Y|X = x_i \quad \sim \quad \left(y^i_1,\ y^i_2,\ \dots,\ y^i_N\right),$$

c) samples size, N, equals to either $100, 500$ or 1000,

d) the standard deviation, σ, takes 14 values distributed between 0.01 and 10.

Moreover, for the Gamma, Normal and Lognormal distributions we assumed that for each input i, the variance of output is the same, i.e.,

$$\text{Var}(Y|X = x_i) = \sigma^2,$$

whereas $\mathbb{E}(Y|X = x_1) = 0$.

On the other hand, for the Exponential distribution, for which variance equals mean squared, we assumed that

$$\text{Var}(Y|X = x_1) = \sigma^2 = \left(\mathbb{E}(Y|X = x_1)\right)^2,$$

and for $i > 1$, as implied by the assumption a) above,

$$\text{Var}(Y|X = x_i) = \left(\mathbb{E}(Y|X = x_i)\right)^2 = \left(\mathbb{E}(Y|X = x_{i-1}) + 1\right)^2.$$

Combinations of different forms of the input distribution, values of N and σ resulted in 168 different settings. For each setting, we have calculated capacity using our method. For a benchmark, we computed true channel capacity by numerical integration of Eq. 2 of the main paper and extensive numerical optimization. Deviations of the capacity calculated with our method from the true capacity as a function of the standard deviation σ are presented in Fig. II.B-E. In the considered 168 different settings the absolute

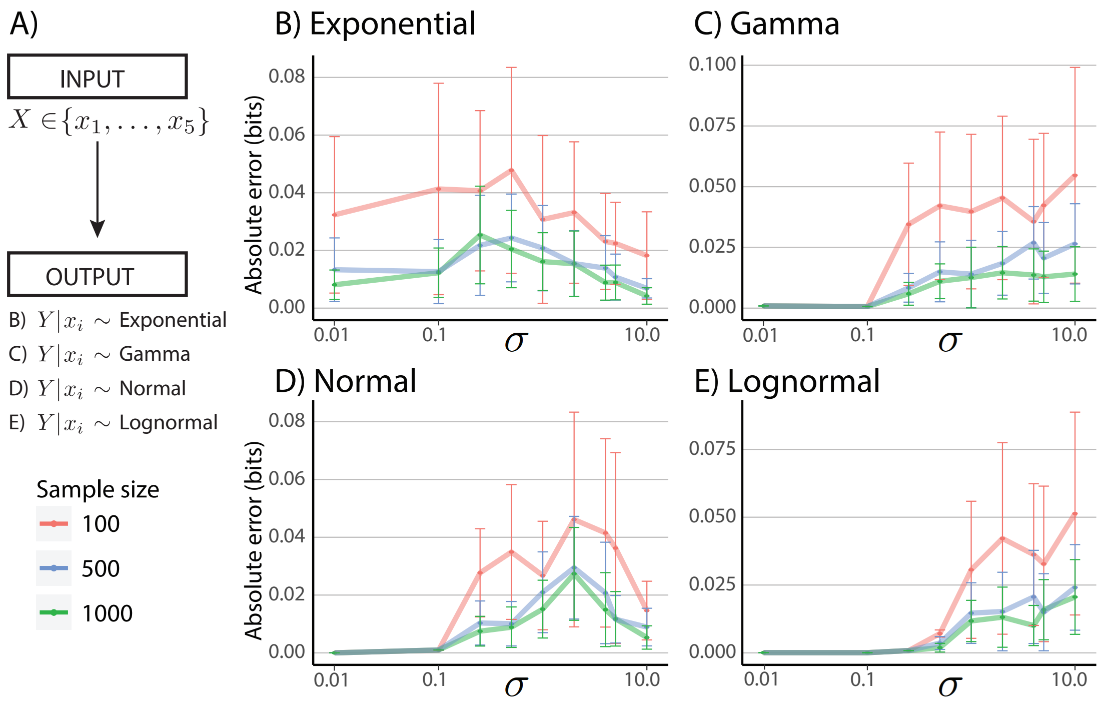

Figure II: Test scenario 3. **(A)** Schematic representation of the test model. **(B-E)** The absolute error of the capacity calculated using our approach with respect to the capacity calculated exactly, i.e., using numerical integration and optimisation. Absolute errors are plotted as a function of σ that controls variance of output distributions. Each panel corresponds to a different type of output distribution, as indicated. Individual lines correspond to different sample size of N. The error-bars show standard deviations of capacity estimates based on 40 repeated samplings.

error of our method did not exceed 0.1 bit and typically was lower than 0.03 bits. We report absolute errors rather than actual capacities compared to the true values as for each level of the standard deviation the capacity may vary according to other factors considered.

The above test example allows us to conclude that our method is robust to the shape of the output distribution, at least as long distributions typical for cellular signaling systems are considered.

3.3 Test scenario 4: toy model of frequency-encoded signals

Previous test scenarios assumed that the input signal modulates the overall level of the output. However, in general, the input signal may change many different properties of the output. For instance, in neuroscience, the stimulus may change the frequency of neural spikes [16, 19, 22]. Also, in the transcriptional regulation higher signal, e.g., the metabolite concentration, may lead to higher frequency of nuclear localisation of a transcription factor [4, 8]. The stimulation level is then considered to be encoded in the frequency of the signaling events. In order to demonstrate the applicability of SLEMI in scenarios, in which the signal changes the frequency of the output events, we have considered the following toy model.

The output $Y(t)$ at any time t is the "off" state, 0, or the "on" state 1 observed with the Gaussian noise, $\mathcal{N}(0, 0.1^2)$. The duration of the "on" state is simplistically assumed to be fixed to 0.3, time units. Depending on the context time units may be different, for instance from seconds in neuroscience, e.g. spikes, to minutes in transcriptional studies. The time between "on" events, denoted here as S_q is assumed to follow the Gamma distribution, typical for waiting times, with mean, and variance, dependent on the input signal, x. Precisely,

$$S_q \sim \text{Gamma}\left(\text{shape} = \frac{1}{\sigma^2},\ \text{scale} = \frac{\sigma^2}{x_i}\right),$$

which implies that

$$\mathbb{E}(S_q) = \frac{1}{x}, \quad \text{Var}(S_q) = \sigma^2 \frac{1}{x^2}.$$

In addition, we considered four values of the input, $x \in \{2^{-5}, 2^{-4}, 2^{-3}, 2^{-2}\}$. Fig. III.A shows schematic representation of the considered toy model. Fig. III.B and III.C show model responses, $Y(t)$, to all four input values (rows) for low and high variance of S_q, respectively.
Further, we considered the output vector, Y, containing 1001 entries representing values of the continuous output, $Y(t)$, at times uniformly distributed between 0 and 100 time units

$$Y = (Y(0), Y(0.1), Y(0.2), \ldots, Y(99.9), Y(100)).$$

Using the output vector, Y, we calculated information capacity as the function of the standard deviation of the duration of the "off" interval, S_q, Fig. III.H. As expected, for low standard deviation the information capacity is close to 2 bits, which is the maximum achievable given the four considered input values. The information capacity decreases with higher values of the standard deviation. Unfortunately, for higher values of the standard deviation, the estimated capacity cannot be easily compared to the true value as, to the best our knowledge, suitable methods are missing. Therefore, what this example demonstrates is that SLEMI can be used to analyze frequency encoded signals, at least from the computational point of view. The accuracy of the approximation is difficult to determine.

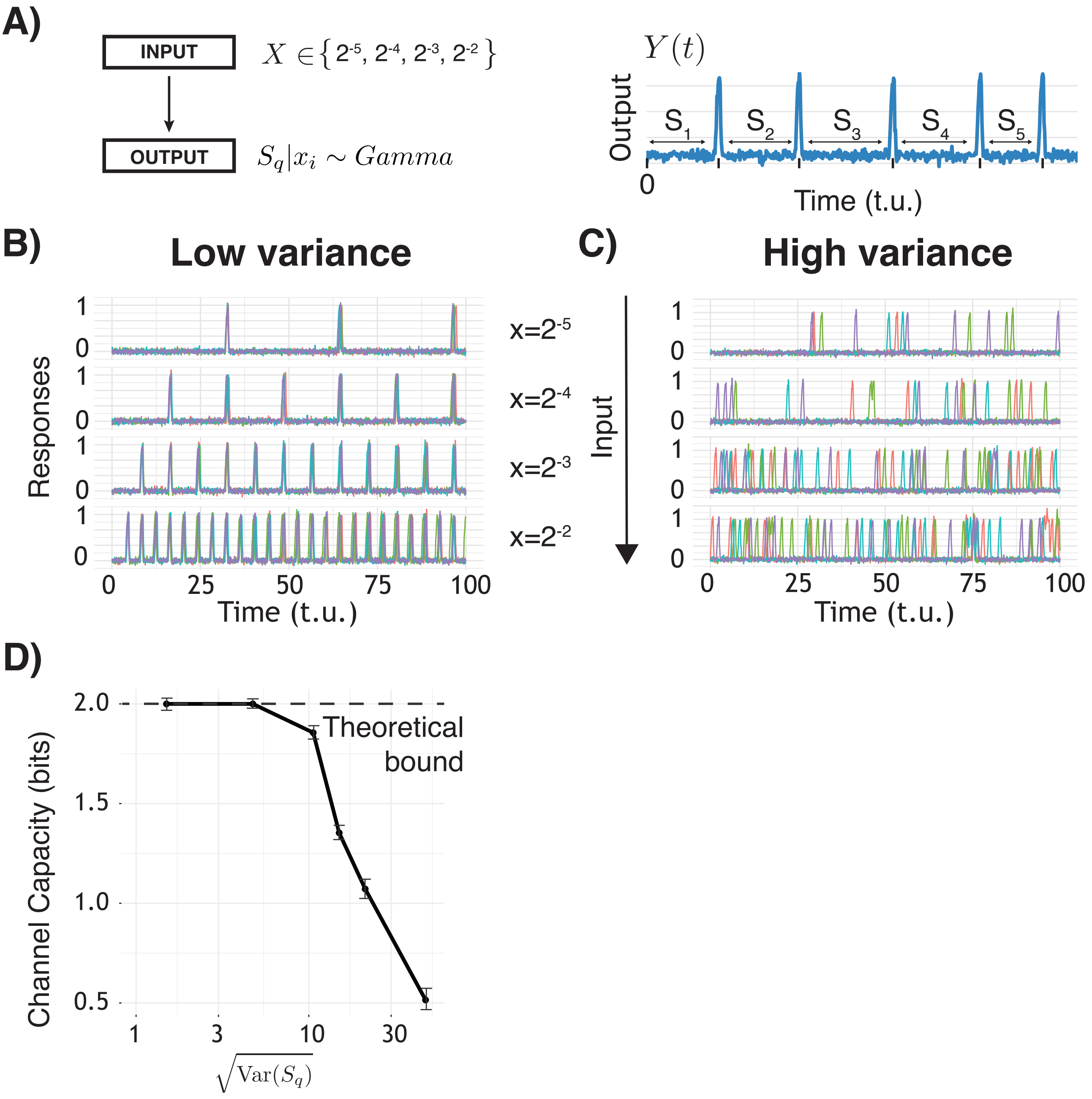

Figure III: Test scenario 4. **(A)** Schematic representation of the considered toy model **(B)** Representative responses, $Y(t)$, from the test model in a low-variance scenario, $\sigma^2 = 0.01$ Individual lines, marked with different colors, correspond to single observations. **(C)** Same as in (B) but for high variance scenario, i.e. $\sigma^2 = 2$. **(H)** Channel capacity as the function standard deviation of the "off" intervals, i.e. $\mathrm{Var}(S_q)^{1/2}$. Theoretical bound (dashed line) indicates a maximal capacity for a channel with 4 inputs (2 bits). Sample sizes in all scenarios are $n = 2000$. The error-bars show standard errors of averages/capacity estimates based on 50 repeated samplings. Capacity was computed for $\sigma^2 \in \{0.01, 0.1, 0.5, 1, 2, 10\}$

4 Analysis of the Nf-κB responses to TNF-α

The NF-κB signaling is one of the key pathways involved in the control of the immune system and one of the first cellular signalling systems studied within the framework of information theory. So far, several papers quantified its information capacity [5, 10, 17, 26]. The main points of interest include whether single-cell responses are switch-like or encode more information about the quantity of stimulus. [5, 17, 26, 20]. In addition, stimuli have been shown to regulate temporal profiles of signaling effectors activities and response dynamics turned out to augment information transmission compared to non-dynamic responses [17, 26]. Given the broad interest to study NF-κB responses within the framework of information theory we have selected the pathway to demonstrate that our framework can indeed provide a novel biological insight.

Broadly speaking, in unstimulated cells the NF-κB protein complex resides in the cytoplasm. Upon activation of the TNF-α receptor, a cascade of events leads to nuclear translocation of NF-κB where it acts as a transcription factor. Activated genes participate in a feedback loop that results in subsequent export of the NF-κB out of the nucleus. The nuclear level of NF-κB is often considered as an immediate output of the system. To examine TNF-α induced NF-κB responses, we have performed a set of experiments in which we stimulated cells with a range of doses of TNF-α. In the examined cell line relA protein, which is a part of the NF-κB complex, was stably fused with a fluorescent dsRed protein. Therefore, life imaging could be used to quantify cytoplasmic and nuclear dsRed fluorescence over time in individual cells. Fluorescence levels were than used as a proxy of cytoplasmic and nuclear levels of NF-κB. The normalised data obtained in the experiments are shown in Fig. IV.C-D. The date were used to perform analysis presented in the Fig. 1 of the main paper. Below we discuss details related to experimental methods, image acquisition and data analysis.

4.1 Experimental methods

We used immortalised murine embryonic fibroblasts cell line (3T3) expressing fluorescent fusion proteins relA-dsRed as wells H2B-GFP for nuclei identification. The cell line was kindly provided by prof. S. Tay and was previously used in several studies, including [20, 10, 21, 13]. Cells were grown and maintained in a conditioned incubator at 37°C, 5 % CO2 in transparent DMEM medium (Life Technologies). As preparation, 48 hours before the experiment, 3T3 cells were resuspended in 3ml of transparent DMEM medium on a confocal dish and keep separately in the incubator. The temperature in the microscope's chamber was set to 37°C and CO2 influx to 5% and 10L. Prior to TNF-α stimulation and imaging. Confocal dish with cultured cells was put in the chamber for an initial phase for about 20 minutes. For NF-α stimulation medium was sucked out from the plate and replaced with a solution of TNF-α and DMEM, which is considered as the start of the experiment (t=0 in the presentation of results). Overall cells were stimulated with 11 different doses of 5-minute pulses of TNF-α (Sigma-Aldrich), ranging from 0 ng/ml to 100 ng/ml. Live imaging was performed using a confocal microscope, Leica TCS SP5 X with an environmental chamber. Channels of dsRed and GFP were used for NF-κB levels and nuclear area identification, respectively. A single imaging experiment lasted 3 hours, during which an image has been captured every 3 minutes both in green (GFP) and red (dsRed) channels, simultaneously at 9 different positions at the plate. Experiment with each concentration of stimulation has been repeated at least four times to test reproducibility and to allow for a sufficient number of observations.

4.2 Image analysis

Images captured every three minutes have been saved as LIF files and then exported to TIFF format using Leica Application Suite 4. TIFF images were segmented to identify nuclear and cytoplasmic areas of individual cells. Individual cells at subsequent images have been tracked to reconstruct cytoplasmic and nuclear fluorescence in individual cells over time. For each stimulation level, we have quantified fluorescence in over 700 hundred cells yielding over 10 000 cells in total. This has been achieved using a customised CellProfiler [9] pipeline as well as manual quality control. Specifically, the developed

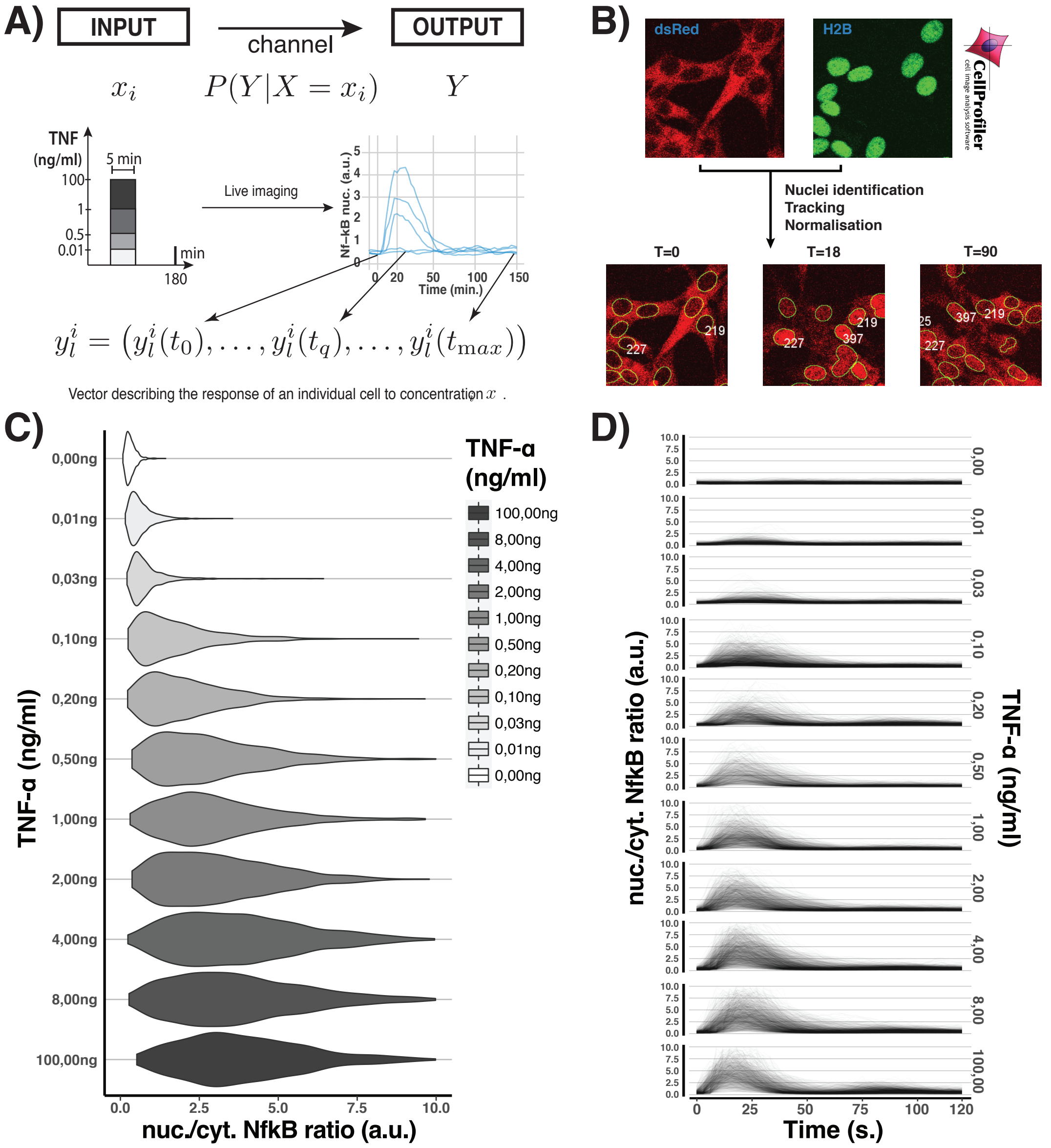

Figure IV: Analysis of the Nf-κB responses to TNF-α. (**A**) Nf-κB responses can be seen as a communication channel. The concentration of TNF-α, x, is considered as input that induces nuclear translocation of the Nf-κB complex. Temporal profile of nuclear Nf-κB level is then considered as output, and the input-output relationship is represented as the probability distribution $P(Y|X = x)$. In experiments, we performed 5-minute pulses of TNF-α stimulation and imaged single cells using confocal microscopy. Images were taken every 3 minutes for 3 hours. (**B**) Acquired images were processed using a customised CellProfiler pipeine. Images were acquired in two channels, corresponding to dsRed and GFP fluorescent proteins. In the examined cell line relA protein, which is a part of the NF-κB complex, was stably fused with a fluorescent dsRed protein. Therefore, life imaging could be used to quantify cytoplasmic and nuclear dsRed fluorescence over time in individual cells. Fluorescence levels were than used as a proxy of cytoplasmic and nuclear levels of NF-κB. In addition,in the studied cell line, the histone protein H2B was fused to the GFP , therefore the GFP fluorescence for nuclear image segmentation. Nuclei were tracked at from 0 to >120 minute with standard algorithm available in CellProfiler. The manipulation of images and data analysis were performed in Python, ImageMagick and R. (**C**) Violin plots of NF-κB responses at 21 min. after stimulation with different doses of TNF-α . (**D**) Temporally resolved responses of single cells (1 line = 1 identified cell) to different doses of TNF-α .

CellProfiler pipeline (available from authors upon request) allowed us to: 1) segment images to identify nuclear area of individual cells (based on GFP fluorescence emitted by histone fluorescent fusion protein H2B-GFP in our cell line); 2) segment cytoplasmic area of individual cells (based on both dsRed and GFP fluorescence); 3) track nuclei between subsequent time frames by simple overlap approach; 4) quantify dsRed fluorescence of the nucleus, $\mathrm{NfKB}_{nuc.}$, in individual cells; 5) quantify dsRed background fluorescence (defined as area not occupied by cells), $\mathrm{NfKB}_{back.}$;
After automated analysis using the above pipeline, we carried out a manual quality control for detection of segmentation and tracking errors. Cytoplasmic Nf-κB , $\mathrm{NfKB}_{cyt.}$ was estimated by enlarging each nucleus by 3 pixels and calculating mean dsRed fluorescence within the ring between enlarged and original nuclei. Next, we subtracted background fluorescence, $\mathrm{NfKB}_{back.}$, both from $\mathrm{NfKB}_{nuc.}$ and $\mathrm{NfKB}_{cyt.}$. To account for photo-bleaching, a simple exponential model was fitted to the time series of the total fluorescence of experimental images and the resulting bleaching factor has been used to scale $\mathrm{NfKB}_{nuc.}$ and $\mathrm{NfKB}_{cyt.}$, respectively. Finally, a ratio $\frac{\mathrm{NfKB}_{nuc.}}{\mathrm{NfKB}_{cyt.}}$ is used as a measure of Nf-κB activation in cells. As the last step, due to small discrepancies in image acquisition times, measurements were interpolated into a unified time interval (from 0 to 120, every 3 minutes) using a simple linear interpolation method. All corrections and normalisation have been conducted in R using custom code and standard packages (available from authors upon request).

4.3 Experimental data set

The data obtained in the experiments can be conceptually represented as

$$y_l^i \quad \sim \quad P(Y|X = x_i), \tag{18}$$

where x_i is the concentration of the 5 minutes pulse TNF-α stimulation, and y_l^i is a vector that describes the response of the l-th cell to stimulation level x_i. Precisely,

$$y_l^i = \left(y_l^i(t_0), ..., y_l^i(t_{\max})\right), \tag{19}$$

where $y_l^i(t_q)$ is the nuclear to cytoplasmic ratio of the l-th cell at time t_q. The data contain responses for 11 different concentrations, i.e. $x_i \in \{0, 0.01, 0.03, 0.1, 0.2, 0.5, 1, 2, 4, 8, 100\}$ ng/ml. The responses were measured every three minutes for two hours. Precisely, $t_0 = 0$, $t_q - t_{q-1} = 3$ and $t_{\max} = 120$. Measured responses are presented in Fig. IV.C and Fig. IV.D.

4.4 Quantification of information capacity

Capacities of time - point responses (Fig. 2B of the main paper) were computed by assuming that the output is composed of individual timepoints

$$y_l^i(t_{\mathrm{q}}), \tag{20}$$

for t_{q} from t_0 to $t_{\max}$. On the other hand, capacities of time - series responses (Fig. 2C of the main paper) were calculated for complete responses truncated at time t_{tr}

$$\left(y_l^i(t_0), \ldots, y_l^i(t_{tr})\right), \tag{21}$$

and calculated capacities for t_{tr} from t_0 to $t_{\max}$.

Uncertainties of the information capacities have been estimated using bootstrap re-sampling. Precisely for each capacity estimate presented in Fig. 2B-C of the main paper, we have computed standard deviation of the capacity calculated 50 times using random 80% of the initial dataset. Grey ribbons denote mean capacity ± standard deviation.

4.5 Redundant information in signaling dynamics

In Fig. 2B-C of the main paper we showed channel capacities of both time-point and time-series responses of the Nf-κB pathway. Recently, in reference [15], authors propose a measure of redundancy of information contained in different entries of the time-series output. Precisely, for a time-series consisting of n time points, $t_1, ..., t_n$, the redundancy measure, MI_{red}, is defined as

$$MI_{\text{red}} = \left(\sum_{q=1}^{n} MI_{t_q} \right) - MI_{\text{TS}}, \tag{22}$$

where MI_{t_q} is the mutual information between the input, X, and the response at the time-point t_q and MI_{TS} is the mutual information between the input, X, and the response at the times t_1,..., t_n. If at each time-point, t_q, unique information, compared to other time points, is transmitted the overall information transmitted will be $\sum_{q=1}^{n} MI_{t_q}$. The information actually transmitted is MI_{TS}, therefore the difference quantifies redundant information, which is a non-negative quantity.
The redundancy is maximal if the same information is transmitted at each time-point is the same. More generally, it can be shown [15] that

$$MI_{\text{red}} \leq (n-1) \left\langle MI_{t_q} \right\rangle, \tag{23}$$

where $\langle MI_t \rangle$ is the average over information transmitted at each time-point, $\langle MI_t \rangle = \frac{1}{n} \sum_{q=1}^{n} MI_{t_q}$.

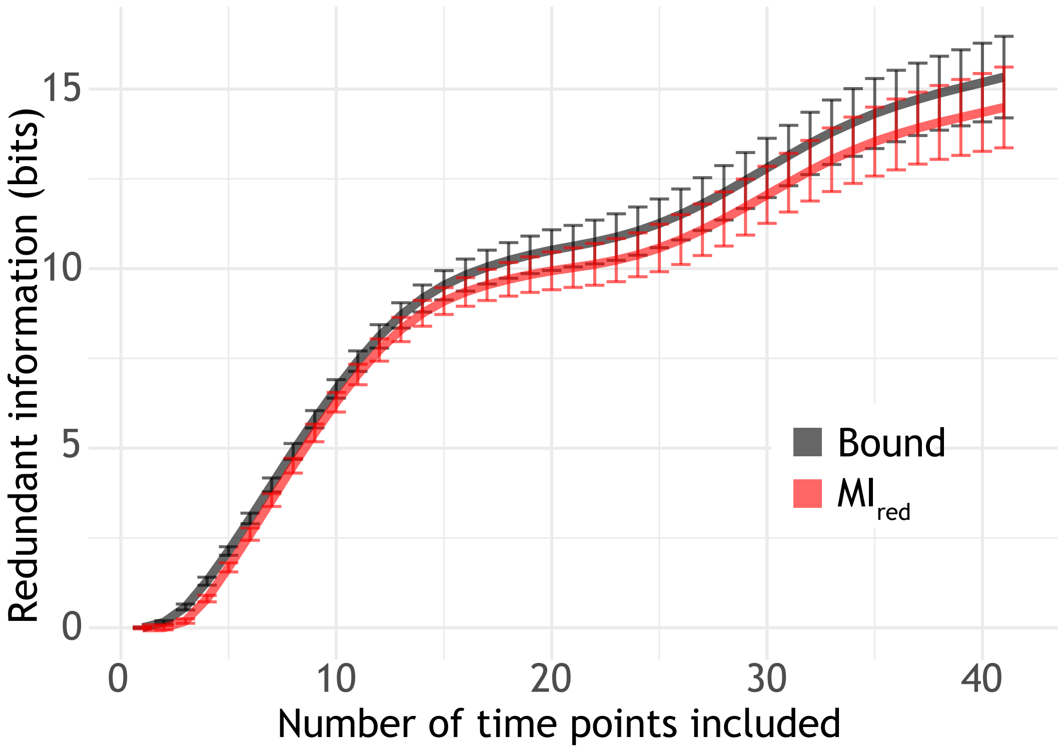

Figure V: Redundant information (red) of Nf-κB pathway calculated from channel capacities of time-point and time-series measurements according to Eq. 22. The bound (black) is the right-hand side of Eq. 23. Errors of estimates were calculated as the sums of errors of information capacity estimates for each time-point obtained with bootstrap

.

Fig. V shows the redundant information as well as its bound calculated for the Nf-κB system. The redundancy is high, and reaches values close to the bound. This is not surprising given the time-point and time-series capacities plotted in Fig. 2B-C of the main paper. The capacity corresponding to time-series, Fig. 2C, increases only moderately at late times, indicating that subsequent time-points carry

only small amount of new information. On the other hand, time-points carry non-zero information, at least around the first and second peak of information transfer, Fig. 2C, which results in high-redundancy systematically summarised in Fig. V.

References

[1] Ian S Abramson. Adaptive density flattening – a metric distortion principle for combating bias in nearest neighbor methods. *The Annals of Statistics*, pages 880–886, 1984.

[2] Suguru Arimoto. An algorithm for computing the capacity of arbitrary discrete memoryless channels. *IEEE Transactions on Information Theory*, 18(1):14–20, 1972.

[3] Richard Blahut. Computation of channel capacity and rate-distortion functions. *IEEE Transactions on Information Theory*, 18(4):460–473, 1972.

[4] Long Cai, Chiraj K Dalal, and Michael B Elowitz. Frequency-modulated nuclear localization bursts coordinate gene regulation. *Nature*, 455(7212):485, 2008.

[5] R Cheong, A Rhee, C J Wang, I Nemenman, and A Levchenko. Information Transduction Capacity of Noisy Biochemical Signaling Networks. *Science*, 334(6054):354–358, 2011.

[6] Thomas M Cover and Joy A Thomas. *Elements of Information Theory.* John Wiley & Sons, 2012.

[7] Shuyang Gao, Greg Ver Steeg, and Aram Galstyan. Efficient estimation of mutual information for strongly dependent variables. In *Artificial Intelligence and Statistics*, pages 277–286, 2015.

[8] Anders S Hansen and Erin K O'Shea. Limits on information transduction through amplitude and frequency regulation of transcription factor activity. *Elife*, 4:e06559, 2015.

[9] Lee Kamentsky, Thouis R Jones, Adam Fraser, Mark-Anthony Bray, David J Logan, Katherine L Madden, Vebjorn Ljosa, Curtis Rueden, Kevin W Eliceiri, and Anne E Carpenter. Improved structure, function and compatibility for CellProfiler: modular high-throughput image analysis software. *Bioinformatics*, 27(8):1179–1180, 2011.

[10] Zbigniew Korwek, Karolina Tudelska, Paweł Nałęcz-Jawecki, Maciej Czerkies, Wiktor Prus, Joanna Markiewicz, Marek Kochańczyk, and Tomasz Lipniacki. Importins promote high-frequency NF-κB oscillations increasing information channel capacity. *Biology Direct*, 11(1):61, 2016.

[11] Alexander Kraskov, Harald Stögbauer, and Peter Grassberger. Estimating mutual information. *Physical Review E*, 69(6):066138, 2004.

[12] Don O Loftsgaarden and Charles P Quesenberry. A nonparametric estimate of a multivariate density function. *The Annals of Mathematical Statistics*, pages 1049–1051, 1965.

[13] Jakub Pękalski, Paweł J Żuk, Marek Kochańczyk, Michael Junkin, Ryan Kellogg, Savaş Tay, and Tomasz Lipniacki. Spontaneous NF-κB activation by autocrine TNF-α signaling: a computational analysis. *PLOS ONE*, 8(11):e78887, 2013.

[14] Barnabás Póczos and Jeff Schneider. On the estimation of α-divergences. In *Proceedings of the Fourteenth International Conference on Artificial Intelligence and Statistics*, pages 609–617, 2011.

[15] Garrett D Potter, Tommy A Byrd, Andrew Mugler, and Bo Sun. Dynamic sampling and information encoding in biochemical networks. *Biophysical journal*, 112(4):795–804, 2017.

[16] Fred Rieke and David Warland. *Spikes: exploring the neural code.* MIT press, 1999.

[17] Jangir Selimkhanov, Brooks Taylor, Jason Yao, Anna Pilko, John Albeck, Alexander Hoffmann, Lev Tsimring, and Roy Wollman. Accurate information transmission through dynamic biochemical signaling networks. *Science*, 346(6215):1370–1373, 2014.

[18] Kumar Sricharan, Raviv Raich, and Alfred O Hero. K-nearest neighbor estimation of entropies with confidence. In *Proceedings of 2011 IEEE International Symposium on Information Theory*, pages 1205–1209, 2011.

[19] Kyle H Srivastava, Caroline M Holmes, Michiel Vellema, Andrea R Pack, Coen PH Elemans, Ilya Nemenman, and Samuel J Sober. Motor control by precisely timed spike patterns. *Proceedings of the National Academy of Sciences*, 114(5):1171–1176, 2017.

[20] Savaş Tay, Jacob J Hughey, Timothy K Lee, Tomasz Lipniacki, Stephen R Quake, and Markus W Covert. Single-cell NF-κB dynamics reveal digital activation and analog information processing in cells. *Nature*, 466(7303):267, 2010.

[21] Savaş Tay, Jake Hughey, Tim K Lee, Markus Covert, and Stephen R Quake. Cells respond digitally to variation in signal intensity via stochastic activation of NF-κB. *Biophysical Journal*, 98(3):429a–430a, 2010.

[22] Frédéric Theunissen and John P Miller. Temporal encoding in nervous systems: a rigorous definition. *Journal of computational neuroscience*, 2(2):149–162, 1995.

[23] Margaritis Voliotis, Rebecca M Perrett, Chris McWilliams, Craig A McArdle, and Clive G Bowsher. Information transfer by leaky, heterogeneous, protein kinase signaling systems. *Proceedings of the National Academy of Sciences*, 111(3):E326–E333, 2014.

[24] Pascal O Vontobel, Aleksandar Kavcic, Dieter M Arnold, and Hans-Andrea Loeliger. A generalization of the Blahut-Arimoto algorithm to finite-state channels. *IEEE Transactions on Information Theory*, 54(5):1887–1918, 2008.

[25] Raymond W Yeung. *Information Theory and Network Coding.* Springer Science & Business Media, 2008.

[26] Qiuhong Zhang, Sanjana Gupta, David L Schipper, Gabriel J Kowalczyk, Allison E Mancini, James R Faeder, and Robin EC Lee. NF-κB dynamics discriminate between TNF-α doses in single cells. *Cell systems*, 5(6):638–645, 2017.
